# Visible-light-induced, Ir-catalyzed reactions of *N*-methyl-*N*-((trimethylsilyl)methyl)aniline with cyclic α,β-unsaturated carbonyl compounds

**DOI:** 10.3762/bjoc.10.86

**Published:** 2014-04-17

**Authors:** Dominik Lenhart, Thorsten Bach

**Affiliations:** 1 Department Chemie and Catalysis Research Center (CRC), Technische Universität München, Lichtenbergstr. 4, D-85747 Garching, Germany, Fax: +49-89-28913315

**Keywords:** cyclization, electron transfer, iridium, photochemistry, photoredox catalysis, radical reactions

## Abstract

*N*-Methyl-*N*-((trimethylsilyl)methyl)aniline was employed as reagent in visible-light-induced, iridium-catalyzed addition reactions to cyclic α,β-unsaturated carbonyl compounds. Typical reaction conditions included the use of one equivalent of the reaction substrate, 1.5 equivalents of the aniline and 2.5 mol % (in MeOH) or 1.0 mol % (in CH_2_Cl_2_) [Ir(ppy)_2_(dtbbpy)]BF_4_ as the catalyst. Two major reaction products were obtained in combined yields of 30–67%. One product resulted from aminomethyl radical addition, the other product was a tricyclic compound, which is likely formed by attack of the intermediately formed α-carbonyl radical at the phenyl ring. For five-membered α,β-unsaturated lactone and lactam substrates, the latter products were the only products isolated. For the six-membered lactones and lactams and for cyclopentenone the simple addition products prevailed.

## Introduction

**Scheme 1:**
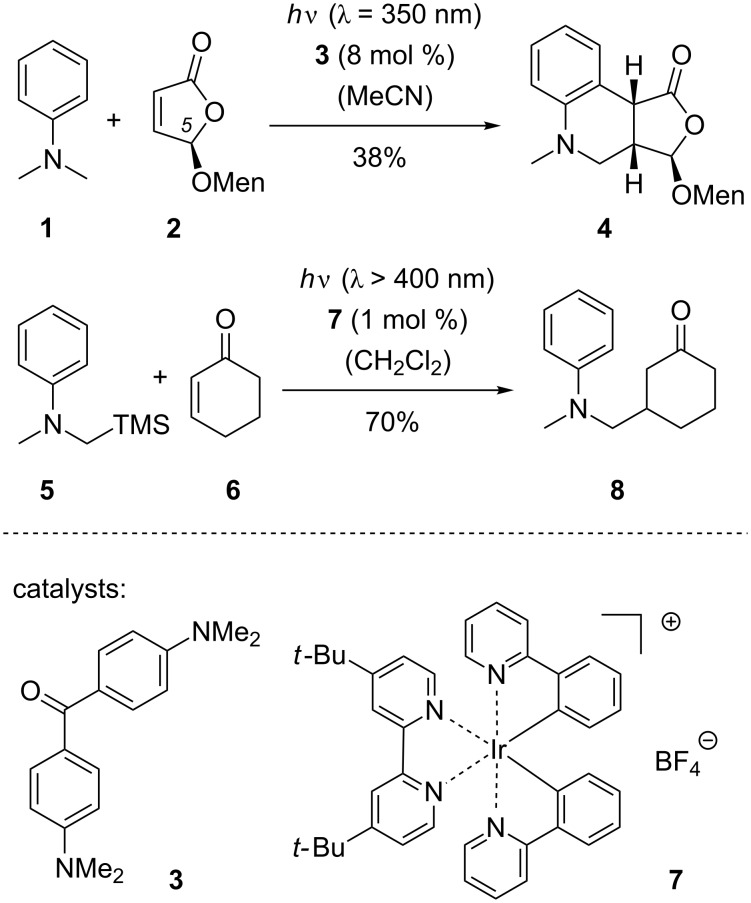
PET-catalyzed addition of *N*,*N*-dimethylaniline (**1**) to furan-2(5*H*)-one **2** [38] and of *N*-methyl-*N*-((trimethylsilyl)methyl)aniline (**5**) to 2-cyclohexenone (**6**) [41].

The photoinduced electron transfer (PET) of an amine to an excited oxidant followed by the loss of a cationic leaving group allows accessing a broad variety of α-aminoalkyl radicals [1–4]. As first shown by Mariano et al. [5–6] and by Pandey et al. [7] a trimethylsilyl (TMS) group is a suitable electrophilic leaving group for this purpose and addition reactions of α-aminoalkyl radicals to double bonds can be induced by irradiation of α-silylated amines in the presence of sensitizers such as 1,4-dicyanonaphthalene or 1,9-dicyanoanthracene. Addition reactions of this type have been broadly used for the formation of carbon–carbon bonds [8–22]. In non-silylated tertiary amines, a proton can act as a leaving group and photoinduced addition reactions of tertiary amines to enones are long known [23–28]. Mechanistically, addition reactions of this type can occur as a radical chain reaction because the addition product of the α-aminoalkyl radical is a carbon-centered radical, which can abstract a hydrogen atom from the tertiary amine [29–30]. Notable contributions to the field of direct tertiary amine addition reactions to enones were made by Hoffmann et al., who established the use of aromatic ketones as suitable PET catalysts for these reactions [31–37]. In Scheme 1, the addition reaction of *N*,*N*-dimethylaniline (**1**) to (5*R*)-menthyloxyfuran-2(5*H*)-one (**2**) is shown, which proceeds to the intriguing tricyclic product **4** employing 4,4’-bis(dimethylamino)benzophenone (**3**) as the catalyst [38]. In this case, the amine was used in large excess (15 equiv) and the chiral menthyl group was derived from (−)-menthol. It was later found that the reaction proceeds with a higher yield (73%) if acetone was employed as a co-solvent [39] and that the diastereoselectivity depends both on the stereogenic center at C5 and on the chirality of the menthyl (Men) backbone [40].

In more recent work [41–42], Nishibayashi et al. showed that iridium complexes can serve as efficient PET catalysts for the addition of α-aminomethyl radicals [43–45] to enones. *N*-Methyl-*N*-((trimethylsilyl)methyl)aniline (**5**) for example served as substrate for the alkylation of 2-cyclohexenone (**6**) employing iridium catalyst **7**. When using the amine as the limiting reagent and an excess of enone (1.5 equiv) product **8** was obtained in 70% yield.

Based on our interest in photochemically induced addition reactions of α-aminoalkyl radicals [46–47], the potential of iridium catalysis attracted our interest and we wondered whether it would be possible to apply the title compound, *N*-methyl-*N*-((trimethylsilyl)methyl)aniline (**5**), in addition reactions to other cyclic α,β-unsaturated carbonyl compounds. In particular, we were interested to see whether cyclization reactions as for product **4** would be observed when using α,β-unsaturated lactones and lactams in combination with a silylated amine and an iridium catalyst. In this article, we provide full details of our studies in this field. Seven different substrates were tested as limiting reagents in the addition reactions and the structure of the respective products was elucidated.

## Results and Dicussion

### Addition to cyclic α,β-unsaturated lactones

**Scheme 2:**
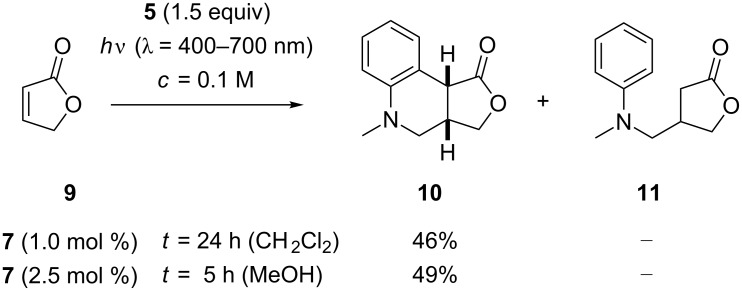
Ir-catalyzed formation of tricyclic product **10** by a domino radical addition reaction to α,β-unsaturated lactone **9**.

Our work commenced with attempted addition reactions to the parent furan-2(5*H*)-one (**9**) and was conducted with a set of eight visible light lamps (Osram L 8W/640 cool white) [48]. A screen of potential catalysts (see Supporting Information File 1 for further details) in CH_2_Cl_2_ as the solvent (*c* = 0.1 M) revealed that [Ir(ppy)_2_(bpy)]BF_4_ (ppy = phenylpyridyl; bpy = 2,2’-bipyridine) and [Ir(ppy)_2_(dtbbpy)]BF_4_ (**7**) (dtbbpy = 4,4’-di-*tert*-butyl-2,2’-bipyridine) gave the best results while other iridium catalysts turned out to be inferior. Remarkably, the desired tricyclic product **10** was isolated as the only product while the direct addition product **11** was not observed. Contrary to that, the ruthenium catalyst Ru(bpy)_3_Cl_2_ delivered a mixture of both products in a ratio of 77/23. Apart from CH_2_Cl_2_, other solvents were tested (see Supporting Information File 1), none of which led to a significant yield improvement. It was observed, however, that the desired reaction was faster in polar solvents such as DMF, DMSO and MeOH. The reaction went to completion in four to five hours while 24 hours were required to reach full conversion in CH_2_Cl_2_. The direct addition product was detected as a side product both in DMF (**10**/**11** = 71/29) and in DMSO (82/18). In MeOH, however, only the tricyclic product **10** was formed. A slight yield improvement was observed when raising the catalyst loading in the latter case from 1 mol % to 2.5 mol % while a decrease of the catalyst concentration led to lower yields. The results of the reactions under optimized conditions are provided for CH_2_Cl_2_ and MeOH as the solvent in Scheme 2. Product **10**, which is a known compound and had been previously prepared by reduction of product **4**, was obtained as a single diastereoisomer [40].

**Scheme 3:**
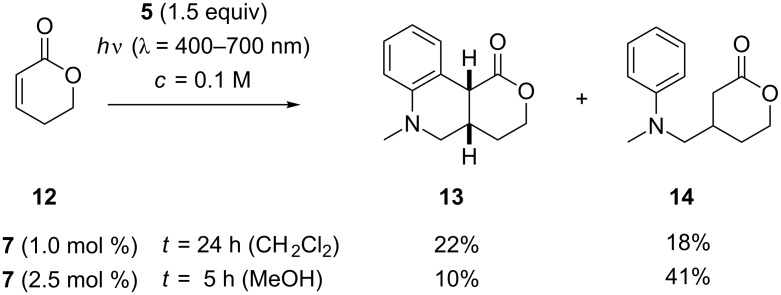
Ir-catalyzed addition reactions of *N*-methyl-*N*-((trimethylsilyl)methyl)aniline (**5**) to 5,6-dihydro-2*H*-pyran-2-one (**12**).

In subsequent sets of experiments the reaction conditions of Scheme 2 were applied to other substrates. With 5,6-dihydro-2*H*-pyran-2-one (**12**) two major products **13** and **14** could be isolated (Scheme 3). If performed in CH_2_Cl_2_ the reaction remained incomplete after 24 hours. The substrate was recovered in 20% and products **13** and **14** were obtained in a ratio of 55/45 with the tricyclic product prevailing. A complete separation of the two compounds was not possible but it could be unambiguously shown that compound **13** was diastereomerically pure. Based on the ^1^H NMR coupling constant (^3^*J*_HH_ = 7.3 Hz) between the two indicated (Scheme 3) protons it was assigned to be the *cis*-diastereoisomer. For the *trans*-diastereoisomer a distinctly larger coupling constant (^3^*J*_HH_ ≥ 10 Hz) would be expected [49]. Primary addition product **14** could be isolated in pure form as it was the predominant reaction product when the reaction was performed in MeOH as the solvent. In this case the reaction went to completion after five hours and delivered product **14** in 41% yield.

### Addition to cyclic α,β-unsaturated ketones

**Scheme 4:**
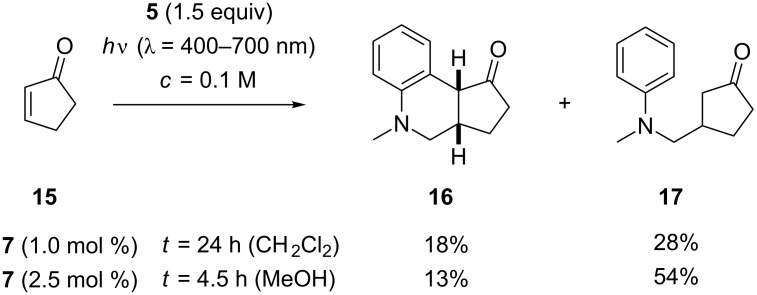
Ir-catalyzed addition reactions of *N*-methyl-*N*-((trimethylsilyl)methyl)aniline (**5**) to 2-cyclopentenone (**15**).

Based on the results obtained with five-membered lactone **9**, it was suprising to note that the related five-membered enone, 2-cyclopentenone (**15**), delivered mainly the direct addition product **17** instead of the tricyclic product **16**. The reactions (Scheme 4) went to completion within 24 hours (in CH_2_Cl_2_) and 4.5 hours (in MeOH). As for compounds **13** and **14**, a complete separation of the two products was not feasible by column chromatography. A mixture of **16** and **17** was obtained, which was free of impurities and in which the relative ratio of products could be established by ^1^H NMR spectroscopy. From a preparative point of view, the result of the reaction in MeOH was more satisfactory because it delivered a combined yield of 67% for both addition products. The relative configuration of compound **16** is tentatively assigned as *cis* due to the coupling constant between the depicted (Scheme 4) protons (^3^*J*_HH_ = 7.0 Hz). The coupling constant is similar to the coupling constant for the known annulated *cis*-product **10** and is in agreement with coupling constants recorded for protons in related tricyclic compounds [50–51].

Replacing 2-cyclopentenone (**15**) by 2-cyclohexenone (**6**) led to the above-mentioned direct addition product **8** (Scheme 1), as previously reported by Nishibayashi et al. [41]. The yield we obtained when using the ketone as the limiting reagent and 1.5 equiv of the amine was 65%. There was no indication for the formation of a tricyclic product.

### Addition to cyclic α,β-unsaturated lactams

**Scheme 5:**
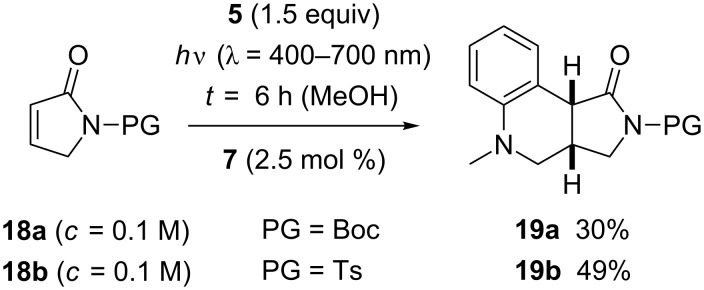
Ir-catalyzed formation of tricyclic products **19** by a domino radical addition reaction to α,β-unsaturated lactams **18**.

Lactams offer the possibility of reactivity tuning by choosing an appropriate protecting group at the nitrogen atom. Since initial experiments with unprotected pyrrolin-2-one were not promising the respective *tert*-butyloxycarbonyl (Boc) and 4-toluenesulfonyl (Ts) derivatives **18a** and **18b** were prepared [52]. In CH_2_Cl_2_ as the solvent, the attempted addition reaction of *N*-methyl-*N*-((trimethylsilyl)methyl)aniline (**5**) turned out to be sluggish. With substrate **18a**, product **19a** was isolated in 23% yield after an irradiation time of 24 hours employing 1 mol % of catalyst **7**. With substrate **18b** the conversion was 64% after 24 hours at an increased catalyst loading of 2.5 mol %. Compound **19b** was isolated in 36% yield (56% based on conversion). Somewhat better results were recorded in MeOH as the solvent (Scheme 5). Irrespective of the protective group, the reaction was complete within six hours and delivered exclusively the tricyclic products **19**. The Boc-protected product **19a** was obtained in only 30% yield, however; the result indicates that the Boc protecting group is not ideal for reactions in MeOH as the solvent (vide infra). A higher yield (49%) was recorded for the Ts-protected product **19b**. The two protons at the ring junction show a similar coupling constant (^3^*J* = 8.1 Hz for **19a**, ^3^*J* = 7.9 Hz for **19b**) as the protons in the related lactone **10** suggesting a *cis*-annulation of the two heterocyclic rings.

**Scheme 6:**
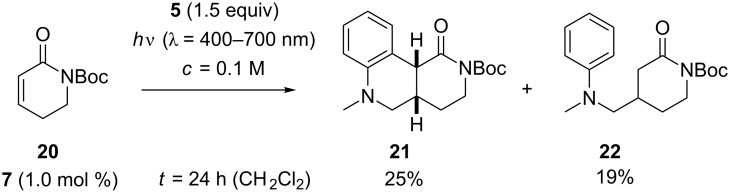
Ir-catalyzed addition reactions of *N*-methyl-*N*-((trimethylsilyl)methyl)aniline (**5**) to α,β-unsaturated lactam **20**.

The instability of Boc-substituted lactams upon irradiation in MeOH solution was also notable in attempted reactions of substrate **20** [53]. With a catalyst loading of 2.5 mol %, the combined yield (10%) of addition products **21** and **22** was disappointingly low at a conversion of 62% after seven hours. The reaction in CH_2_Cl_2_ proceeded more smoothly and delivered after an irradiation time of 24 hours the two products **21** and **22** in a combined yield of 44% (Scheme 6). There was no yield improvement if the catalyst loading was increased to 2.5 mol %.

**Scheme 7:**
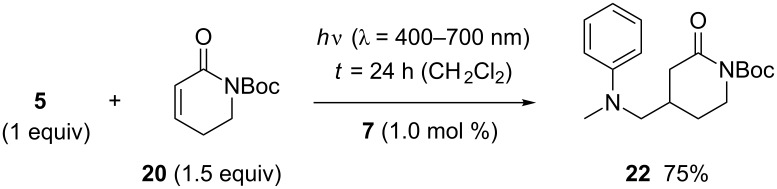
Ir-catalyzed addition reactions of *N*-methyl-*N*-((trimethylsilyl)methyl)aniline (**5**) to α,β-unsaturated lactam **20** with the aniline being the limiting substrate.

With an excess of α,β-unsaturated lactam **20**, the reaction turned out to proceed with higher type selectivity. The domino process was suppressed and the plain addition product **22** was obtained in 75% yield (Scheme 7).

The latter result suggested that the previously discussed (Scheme 3) addition reaction to 5,6-dihydro-2*H*-pyran-2-one (**12**) might also lead to a single product if performed with the aniline as the limiting agent. To our surprise, however, the relative product ratio remained unchanged when the substrate ratio **5**/**12** = 1.5/1 was decreased to 1/1.5.

**Scheme 8:**
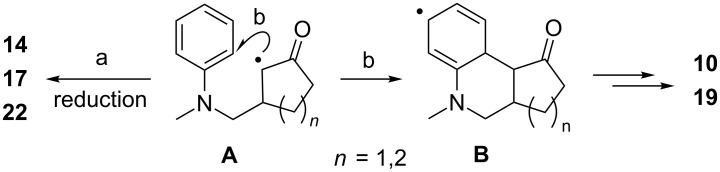
Cyclization of putative radical **A** to intermediate **B** competes with reduction of **A** to form addition products, such as **14**, **17**, **22**.

Mechanistically, it is likely that the well-established photoredox cascade between aniline **5** and the photoexcited iridium complex **7** operates [54–58]. Oxidation of the aniline and loss of the trimethylsilyl group leads to an α-aminomethyl radical, which adds to the cyclic α,β-unsaturated carbonyl compound (Scheme 8). The intermediate radical **A** can undergo immediate reduction presumably by concomitant oxidation of the previously reduced iridium complex (path a), which provides the simple addition products (e.g., **14**, **17**, **22**) [41–42], or it attacks (path b) the adjacent phenyl group to form radical **B**. The further fate of radical **B** is likely an oxidation to the tricyclic products (e.g., **10**, **19**). The oxidation could occur by hydrogen transfer to the starting material, i.e., the α,β-unsaturated carbonyl compound [39], which would explain why the yields never exceeded 50%. Upon hydrogen transfer the α,β-unsaturated carbonyl compound would give a cyclic α-acyl radical, which would be capable to re-oxidize the reduced iridium complex thus completing the catalytic cycle. However, we have not been able to substantiate the latter hypothesis, e.g., by isolation of a saturated carbonyl compound.

Interestingly, we did not find a hint for a radical chain pathway, which would include hydrogen abstraction from the starting aniline **5** by intermediate **A**. The resulting radical would carry a trimethylsilyl group, which would be eventually found in the product. The absence of a trimethylsilyl group in the products indicates that reduction (path a) and cyclization (path b) are more efficient than hydrogen abstraction while hydrogen abstraction has been established as a radical chain propagating step in reactions performed with UV light [5–6,8,11,32,34,38–40].

## Conclusion

In summary, it was shown that a photochemical generated aminomethyl radical – produced from *N*-methyl-*N*-((trimethylsilyl)methyl)aniline – adds readily not only to the previously reported cyclohexenone but to several cyclic α,β-unsaturated carbonyl compounds. Further reaction of the intermediate α-carbonyl radical was observed with five-membered substrates leading to synthetically interesting tricyclic products. The latter reaction suffers from the fact that oxidation of the putative intermediate **B** is required, which seems to occur at the expense of the substrate. If a suitable compound was found to adapt the role of an ancillary oxidant, yields could possibly be improved.

## Supporting Information

File 1 Experimental section.

File 2 Tables of all optimization experiments and copies of ^1^H/^13^C spectra of PET catalysis products.
