# Supplementary material for: Visible-light-induced, Ir-catalyzed reactions of N-methyl-N-((trimethylsilyl)methyl)aniline with cyclic α,β-unsaturated carbonyl compounds
Source: Beilstein J Org Chem. 2014 Apr 17;10:890–6. doi: 10.3762/bjoc.10.86 (PMC3999855; doi:10.3762/bjoc.10.86)
Supplement: File 1 — Experimental section. [file Beilstein_J_Org_Chem-10-890-s001.pdf]

Supporting Information File 1

for

Visible-light-induced, Ir-catalyzed reactions of *N*-methyl-*N*-((trimethylsilyl))methylaniline with cyclic α,β-unsaturated carbonyl compounds

Dominik Lenhart and Thorsten Bach*

Address: Department Chemie and Catalysis Research Center (CRC), Technische Universität München, Lichtenbergstr. 4, D-85747 Garching, Germany, Fax: +49-89-28913315

Email: Thorsten Bach* - thorsten.bach@ch.tum.de

* Corresponding author

Experimental section

Contents

• 1. Experimental ••S2

• 2. References ••S11

1. Experimental

General: Reactions containing moisture sensitive compounds were conducted in flame-dried glass vessels under argon atmosphere. Dry solvents (CH_3CN, MeOH, DMF, toluene, 1,4-dioxane, DMSO, DMA) were purchased from *Sigma-Aldrich*, *Acros Organics, Fluka* or *Merck*. Dry CH_2Cl_2 and THF were taken from a MB-SPS-800 apparatus (*M. Braun*). Common solvents for chromatography [pentane (P), EtOAc, CH_2Cl_2, MeOH] were distilled prior to use. IR spectra were recorded on a *JASCO* IR-4100 (ATR); MS and HRMS measurements were performed on a *Finnigan* MAT 8200 (EI), a *Finnigan* MAT 95-S (HR–EI), a *Finnigan* LCQ classic (ESI) and a Thermo *Finnigan* LTQ FT (HRMS–ESI). ^1H and ^{13}C NMR: Bruker AV-250, Bruker AV-360, Bruker AV-500, Bruker AV-500cr recorded at 303 K. Chemical shifts are reported relative to the used solvent ($CHCl_3$). All coupling constants (*J*) are reported in Hertz (Hz). Melting points were measured on a *Kofler* melting point apparatus (*Reichert*) and are uncorrected.

Photoreactions were carried out in Pyrex phototubes (diameter = 1 cm) using 8 *Osram* cool white lamps (L 8W/640) [S1]. α,β-Unsaturated compounds **9**, **12** and **15** were purchased from *Alfa Aesar* and *Sigma-Aldrich*. Compounds **18a** [S2], **18b** [S2], and **20** [S3] were prepared according to literature known procedures. Amine **5** was synthesized from *N*-methylaniline [S4]. Photocatalysts $[Ir(ppy)_2(bpy)]BF_4$ [S5], $[Ir(ppy)_2(dtbbpy)]BF_4$ (**7**) [S5], $[Ir((dF)(CF_3)\text{-}ppy)_2(bpy)]PF_6$ [S6], $[Ir((dF)(CF_3)ppy)_2(dtbbpy)]PF_6$ [S6] were prepared by heating of the corresponding iridium dimer with the according ligand in ethylene glycol.

General procedure for PET catalyzed reactions

In a flame-dried phototube, all compounds except the photocatalyst were dissolved in the corresponding solvent under an argon atmosphere. The solution was degassed three times via freeze–pump–thaw cycles prior to addition of the catalyst. Irradiation was followed by

addition of 10 mL saturated aqueous $NaHCO_3$ solution and 15 mL CH_2Cl_2. The layers were separated and the aqueous layer was extracted with CH_2Cl_2 (2 × 10 mL). The combined organic layers were dried over Na_2SO_4, filtered and the solvent was removed under reduced pressure. The residue was purified by flash chromatography on silica.

5-Methyl-3a,4,5,9b-tetrahydrofuro[3,4-*c*]quinolin-1(3*H*)-one (10)

According to the general procedure for PET reactions, 7.17 μL (8.58 mg, 100 μmol, 1.00 equiv) of **9** and 32.6 μL (29.0 mg, 150 μmol, 1.50 equiv) of **5** were irradiated for 5 h (MeOH) or 24 h (CH_2Cl_2) in 1 mL of the corresponding solvent. In CH_2Cl_2 1 mol % of catalyst **7** (856 μg) were used, in MeOH 2.5 mol % (2.14 mg). Column chromatography on silica (P/EtOAc 1:1) gave for the reaction in CH_2Cl_2 9.4 mg (46 μmol, 46%) for the reaction in MeOH 10.0 mg (49 μmol, 49%) of the title compound as a white solid.

R_f = 0.50 (P/EtOAc 1:1) [UV, $KMnO_4$].

^{1}H-NMR (500 MHz, $CDCl_3$): δ (ppm) = 2.89 (s, 3 H, *N*-CH_3), 2.92 (dd, ^2J = 11.6 Hz, ^3J = 8.8 Hz, 1 H, C4-H), 3.08 (virt. tddd, ^3J ≅ 8.4 Hz, ^3J = 6.7 Hz, ^3J = 4.6 Hz, ^3J = 3.6 Hz, 1 H, C3a-H), 3.22 (ddd, ^2J = 11.6 Hz, ^3J = 4.6 Hz, ^4J = 0.8 Hz, 1 H, C4-H), 3.71 (d, ^3J = 8.4 Hz, 1 H, C9b-H), 4.21 (dd, ^2J = 9.2 Hz, ^3J = 3.6 Hz, 1 H, C3-H), 4.47 (dd, ^2J = 9.2 Hz, ^3J = 6.7 Hz, 1 H, C3-H), 6.71 (dd, ^3J = 8.4 Hz, ^4J = 1.0 Hz, 1 H, C6-H), 6.83 (td, ^3J = 7.5 Hz, ^4J = 1.0 Hz, 1 H, C8-H), 7.19-7.22 (m, 1 H, C7-H), 7.47 (d, ^3J = 7.5 Hz, 1 H, C9-H).

^{13}C-NMR (90.6 MHz, $CDCl_3$): δ (ppm) = 34.3 (C3a), 39.7 (CH_3), 40.9 (C9b), 51.3 (C4), 69.6 (C3), 112.3 (C6), 116.9 (C9a), 118.5 (C8), 128.7 (C7), 130.7 (C9), 146.9 (C5a), 176.8 (C1).

The analytical data are in agreement with the previously reported data for this compound [S7].

6-Methyl-3,4,4a,5,6,10b-hexahydro-1*H*-pyrano[4,3-*c*]quinolin-1-one (13) and 4-{[methyl(phenyl)amino]methyl}tetrahydro-2*H*-pyran-2-one (14)

According to the general procedure for PET reactions, 22.7 μL (25.8 mg, 250 μmol, 1.00 equiv) of **12** and 81.3 μL (72.4 mg, 375 μmol, 1.50 equiv) of **5** were irradiated for 5 h (MeOH) or 24 h (CH_2Cl_2) in 2.5 mL of the corresponding solvent. In CH_2Cl_2 1 mol % of catalyst **7** (2.14 mg) were used, in MeOH 2.5 mol % (5.35 mg). Column chromatography on silica (P/EtOAc 2:1) yielded for the reaction in MeOH 27.6 mg product, for the reaction in CH_2Cl_2 22.3 mg. The relative amounts of **13** and **14** were determined by ^{1}H NMR integration (MeOH reaction **13**/**14** = 20:80, CH_2Cl_2 reaction: **13**/**14** = 55:45).

R_f = 0.39 (**14**), 0.34 (**13**) (P/EtOAc 1:1) [UV, $KMnO_4$].

IR (ATR): $\tilde{\upsilon}$ = 3061 cm^{-1}, 3025, 2906, 2827, 1729, 1599, 1505, 1364, 1256, 1079, 992, 747.

6-Methyl-3,4,4a,5,6,10b-hexahydro-1*H*-pyrano[4,3-*c*]quinolin-1-one (13)

^{1}H-NMR (500 MHz, $CDCl_3$): δ (ppm) = 1.59-1.67 (m, 1 H, C4-H), 1.71-1.79 (m, 1 H, C4-H), 2.76-2.84 (m, 1 H, C4a-H), 2.93 (s, 3 H, CH_3), 2.98 (dd, ^2J = 11.6 Hz, ^3J = 8.5 Hz, 1 H, C5-H), 3.23 (ddd, ^2J = 11.6 Hz, ^3J = 3.5 Hz, J = 0.8 Hz, 1 H, C5-H), 3.95 (d, ^3J = 7.3 Hz, 1 H, C10b-H), 4.27 (ddd, ^2J = 11.4 Hz, ^3J = 7.3 Hz, J = 0.8 Hz, 1 H, C3-H), 4.35 (ddd, ^2J = 11.4 Hz, ^3J = 8.8 Hz, ^3J = 4.3 Hz, 1 H, C3-H), 6.68-6.74 (m, 2 H, C8-H, C10-H), 7.18 (d, ^3J = 7.6 Hz, 1 H, C7-H), 7.21 (td, ^3J = 8.3 Hz, ^4J = 1.4 Hz, 1 H, C9-H).

^{13}C-NMR (126 MHz, $CDCl_3$): δ (ppm) = 26.3 (C4), 29.1 (C4a), 39.6 (CH_3), 43.0 (C10b), 55.2 (C5), 66.3 (C3), 112.1 (C6), 116.5 (s. C10a), 117.6 (C8), 128.9 (C7), 130.4 (C9), 146.4 (C6a), 173.0 (C1).

MS (EI): *m/z* (%) = 217 [M^+] (9), 158 (1), 145 (3), 120 (100), 106 (6), 91 (2), 76 (8).

HRMS (EI): calc. for $C_{13}H_{15}NO_2$ = 217.1097, found: 217.1095.

4-{[Methyl(phenyl)amino]methyl}tetrahydro-2*H*-pyran-2-one (14)

IR (ATR): ῡ = 3061 cm^{-1}, 3025, 2906, 2827, 1729, 1599, 1505, 1364, 1256, 1079, 992, 747.

^{1}H-NMR (500 MHz, $CDCl_3$): δ (ppm) = 1.58-1.67 (m, 1 H, C5-H), 1.99-2.05 (m, 1 H, C5-H), 2.26 (dd, ^2J = 17.2 Hz, ^3J = 10.1 Hz, 1 H, C3-H), 2.45-2.55 (m, 1 H, C4-H), 2.72 (ddd, ^2J = 17.2 Hz, ^3J = 6.1 Hz, ^4J = 1.5 Hz, 1 H, C3-H), 3.01 (s, 3 H, CH_3), 3.24-3.33 (m, 2 H, *N*-CH_2), 4.26 (td, ^2J = 11.3 Hz, ^3J = 3.7 Hz, 1 H, C6-H), 4.46 (ddd, ^2J = 11.3 Hz, ^3J = 4.9 Hz, ^3J = 3.7 Hz, 1 H, C6-H), 6.72 (dd, ^3J = 8.7 Hz, ^4J = 0.8 Hz, 2 H, C_{ortho}-H), 6.76 (tt, ^3J = 7.3 Hz, ^4J = 0.8 Hz, 1 H, C_{para}-H), 7.25-7.29 (m, 2 H, C_{meta}-H).

^{13}C-NMR (90.6 MHz, $CDCl_3$): δ (ppm) = 27.3 (C5), 31.0 (C4), 34.4 (C3), 40.0 (CH_3), 58.2 (*N*-CH_2), 68.6 (C6), 112.5 (2 × C_{ortho}), 117.2 (C_{para}), 129.5 (2 × C_{meta}), 149.3 (C_{ar}-*N*), 170.9 (C2).

MS (EI): *m/z* (%) = 219 [M^+] (13), 174 [$M^+ - CO_2$] (5), 135 (19), 120 (100), 106 (31), 93 (20), 85 (41), 83 (63).

HRMS (EI): calc. for $C_{13}H_{17}NO_2$ = 219.1254, found: 219.1254.

5-Methyl-2,3,3a,4,5,9b-hexahydro-1*H*-cyclopenta[*c*]quinolin-1-one (16) and 3-{[methyl(phenyl)amino]methyl}cyclopentan-1-one (17)

According to the general procedure for PET reactions, 21.4 µL (20.9 mg, 250 µmol, 1.00 equiv) of **15** and 81.3 µL (72.4 mg, 375 µmol, 1.50 equiv) of **5** were irradiated for 4.5 h (MeOH) or 24 h (CH_2Cl_2) in 2.5 mL of the corresponding solvent. In CH_2Cl_2 1 mol % of catalyst **7** (2.14 mg) were used, in MeOH 2.5 mol % (5.35 mg). Column chromatography on silica (P/EtOAc 15:1→ 10:1) yielded 34.1 mg product for the reaction in MeOH and 23.0 mg for CH_2Cl_2. Relative amounts of **16** and **17** were determined by ^{1}H NMR integration. (MeOH reaction **16**/**17** = 19:81, CH_2Cl_2 reaction: **16**/**17** = 39:61)

R_f = 0.56 (**16**), 0.57 (**17**) (P/EtOAc 1:1) [UV, $KMnO_4$].

IR (ATR): $\tilde{\upsilon}$ = 3094 cm^{-1}, 3061, 3025, 2952, 2935, 2898, 2877, 1739, 1599, 1505, 1360, 1155, 992, 747.

5-Methyl-2,3,3a,4,5,9b-hexahydro-1*H*-cyclopenta[*c*]quinolin-1-one (16)

^{1}H-NMR (500 MHz, $CDCl_3$): δ (ppm) = 1.97-2.03 (m, 1 H, C3-H), 2.11-2.16 (m, 1 H, C3-H), 2.31-2.37 (m, 2 H, C2-H), 2.82-2.87 (m, 2 H, C3a-H, C4-H), 2.99 (s, 3 H, CH_3), 3.20 (*virt.* q, $^3J \cong$ 8.5 Hz, 1 H, C4-H), 3.31 (d, ^3J = 7.0 Hz, 1 H, C9b-H), 6.67 (d, ^3J = 8.4 Hz, 1 H, C6-H), 6.77 (td, ^3J = 7.5 Hz, ^4J = 0.8 Hz, 1 H, C8-H), 7.13-7.17 (m, 1 H, C7-H), 7.34 (d, ^3J = 7.5 Hz, 1 H, C9-H).

^{13}C-NMR (126 MHz, $CDCl_3$): δ (ppm) = 24.1 (C3), 34.7 (C3a), 34.8 (C2), 39.8 (CH_3), 50.0 (C9b), 52.9 (C4), 111.9 (C6), 117.8 (C8), 117.8 (C9a), 128.0 (C7), 130.7 (C9), 146.4 (C5a), 217.1 (C1).

MS (ESI): *m/z* (%) = 202 [M^+ + H] (100), 184 [M^+ − OH] (15), 158 (4), 111 (21), 78 (1).

HRMS (ESI): calc. for $C_{13}H_{16}NO$ = 202.12264, found: 202.12269.

3-{[Methyl(phenyl)amino]methyl}cyclopentan-1-one (17)

^{1}H-NMR (500 MHz, $CDCl_3$): δ (ppm) = 1.61-1.72 (m, 1 H, C4-H), 1.95 (ddd, ^2J = 18.4 Hz, ^3J = 10.1 Hz, ^4J = 1.2 Hz, 1 H, C2-H), 2.13-2.21 (m, 2 H, C4-H, C5-H), 2.30-2.36 (m, 1 H, C5-H), 2.38 (dd, ^2J = 18.4 Hz, ^3J = 7.3 Hz, 1 H, C2-H), 2.61-2.71 (m, 1 H, C3-H), 2.98 (s, 3 H, CH_3), 3.40 (d, ^3J = 7.1 Hz, 2 H, *N*-CH_2), 6.71-6.73 (m, 3 H, C_{para}-H, C_{ortho}-H), 7.23-7.26 (m, 2 H, C_{meta}-H).

^{13}C-NMR (90.6 MHz, $CDCl_3$): δ (ppm) = 27.8 (C4), 36.2 (C3), 38.3 (C5), 39.6 (CH_3), 43.6 (C2), 57.5 (*N*-CH_2), 112.4 (2 × C_{ortho}), 116.8 (C_{para}), 129.5 (2 × C_{meta}), 149.4 (C_{ar}-*N*), 218.7 (C1).

MS (ESI): *m/z* (%) = 204 [M^+ + H] (57), 184 (4), 120 (5), 111 (100).

HRMS (ESI): calc. for $C_{13}H_{18}NO$ = 204.13829, found: 204.13836.

***tert*-Butyl 5-methyl-1-oxo-1,3,3a,4,5,9b-hexahydro-2*H*-pyrrolo[3,4-*c*]quinoline-2-carboxylate (19a)**

According to the general procedure for PET reactions, 45.8 mg (250 μmol, 1.00 equiv) of **18a** and 81.3 μL (72.4 mg, 375 μmol, 1.50 equiv) of **5** were irradiated for 6 h in 2.5 mL of MeOH using 2.5 mol % (5.35 mg) of catalyst **7**. Column chromatography on silica (P/EtOAc 5:1 → 3:1) yielded 22.7 mg (75.0 μmol, 30%) of **19a** as pale yellow oil. R_f = 0.54 (P/EtOAc 1:1) [UV, $KMnO_4$].

IR (ATR): $\tilde{\upsilon}$ = 3068 cm^{-1}, 3032, 2978, 2931, 2902, 2811, 1779, 1747, 1710, 1602, 1501, 1368, 1151.

^{1}H-NMR (500 MHz, $CDCl_3$): δ (ppm) = 1.51 (s, 9 H, $C(CH_3)_3$), 2.82-2.87 (m, 1 H, C3a-H), 2.88 (s, 3 H, *N*-CH_3), 2.90-2.94 (m, 1 H, C3-H), 3.18 (dd, ^2J = 11.0 Hz, ^3J = 3.6 Hz, 1 H, C3-H), 3.66 (dd, ^2J = 11.0 Hz, ^3J = 3.9 Hz, 1 H, C4-H), 3.70 (d, ^3J = 8.1 Hz, 1 H, C9b-H), 3.88 (dd, ^2J = 11.0 Hz, ^3J = 7.1 Hz, 1 H, C4-H), 6.68 (dd, ^3J = 8.3 Hz, ^4J = 1.0 Hz, 1 H, C6-H), 6.80 (td, ^3J = 7.5 Hz, ^4J = 1.0 Hz, 1 H, C8-H), 7.17 (ddd, ^3J = 8.3 Hz, ^3J = 7.5 Hz, ^4J = 0.8 Hz, 1 H, C7-H), 7.47 (d, ^3J = 7.5 Hz, 1 H, C9-H).

^{13}C-NMR (90.6 MHz, $CDCl_3$): δ (ppm) = 28.3 (3 × CH_3), 29.8 (C3a), 39.7 (*N*-CH_3), 45.1 (C9b), 48.1 (C4), 51.9 (C3), 83.3 ($\underline{C}(CH_3)_3$), 112.0 (C6), 117.5 (C9a), 118.2 (C8), 128.5 (C7), 130.9 (C9), 146.9 (C5a), 151.0 (N$\underline{C}$OO), 173.0 (C1).

MS (ESI): *m/z* (%) = 303 [M^+ + H] (67), 247 (100), 203 (58).

HRMS (ESI): calc. for $C_{17}H_{23}N_2O_3$ = 303.1709, found: 303.1707.

5-Methyl-2-tosyl-2,3,3a,4,5,9b-hexahydro-1*H*-pyrrolo[3,4-*c*]quinolin-1-one (19b)

According to the general procedure for PET reactions, 47.4 mg (200 μmol, 1.00 equiv) of **19a** and 65.1 μL (57.9 mg, 300 μmol, 1.50 equiv) of **5** were irradiated for 5 h in 2 mL of MeOH using 2.5 mol % (4.28 mg) of catalyst **7**. Column chromatography on silica (P/EtOAc 7:3) yielded 34.9 mg (98.0 μmol, 49%) of **19b** as off white solid. R_f = 0.22 (P/EtOAc 7:3) [UV, $KMnO_4$]; mp: 155 °C.

IR (ATR): $\tilde{\upsilon}$ = 3036 cm^{-1}, 2992, 2949, 2924, 2873, 2808, 2757, 1728, 1678, 1602, 1509, 1201, 952.

^{1}H-NMR (500 MHz, $CDCl_3$): δ (ppm) = 2.41 (s, 3 H, CH_3), 2.75-2.80 (m, 1 H, C3-H), 2.84 (s, 3 H, *N*-CH_3), 2.89 (qdd, ^3J = 8.0 Hz, ^3J = 4.5 Hz, ^3J = 2.9 Hz, 1 H, C3a-H), 3.15 (ddd, ^2J = 11.5 Hz, ^3J = 4.5 Hz, ^4J = 0.7 Hz, 1 H, C3-H), 3.63 (d, ^3J = 8.0 Hz, 1 H, C9b-H), 3.77 (dd, ^2J = 10.3 Hz, ^3J = 2.9 Hz, 1 H, C4-H), 4.05 (dd, ^2J = 10.3 Hz, ^3J = 6.9 Hz, 1 H, C4-H), 6.65 (d, ^3J = 8.2 Hz, 1 H, C6-H), 6.74 (td, ^3J = 7.3 Hz, ^4J = 0.7 Hz, 1 H, C8-H), 7.15 (ddd, ^3J = 8.2 Hz, ^3J = 7.3 Hz, ^4J = 1.6 Hz, 1 H, C7-H), 7.28-7.31 (m, 3 H, C9-H, C_{meta}-H), 7.88 (d, ^3J = 8.2 Hz, 2 H, C_{ortho}-H).

^{13}C-NMR (90.6 MHz, $CDCl_3$): δ (ppm) = 21.9 (CH_3), 30.4 (C3a), 39.6 (*N*-CH_3), 44.5 (C9b), 48.9 (C4), 51.7 (C3), 112.1 (C6), 116.3 (C9a), 118.2 (C8), 128.3 (2 × C_{ortho}), 128.7 (C7), 129.9 (2 × C_{meta}), 130.8 (C9), 135.2 (C-S), 145.4 (<u>C</u>-CH_3), 146.7 (C5a), 172.4 (C1).

MS (EI): *m/z* (%) = 356 [M^+] (3), 279 (4), 234 (58), 171 (19), 135 (54), 106 (60), 57 (100).

HRMS (EI): calc. for $C_{19}H_{20}N_2O_3S$ = 356.1189, found: 356.1187.

***tert*-Butyl 4-{[methyl(phenyl)amino]methyl}-2-oxopiperidine-1-carboxylate (22)**

According to the general procedure for PET reactions, 54.3 µL (48.3 mg, 250 µmol, 1.00 equiv) of **5** and 69.1 µL (73.9 mg, 375 µmol, 1.50 equiv) of **20** were irradiated for 24 h in 2.5 mL of CH_2Cl_2 using 1 mol % of catalyst **7** (2.14 mg). Column chromatography on silica (P/EtOAc 4:1) yielded 60.0 mg (188 µmol, 75%) of **22** as pale yellow oil. R_f = 0.15 (P/EtOAc 4:1) [UV, $KMnO_4$].

IR (ATR): ῦ = 2978 cm^{-1}, 2931, 2826, 1768, 1707, 1598, 1505, 1476, 1451, 1393, 1368.

^{1}H-NMR (500 MHz, $CDCl_3$): δ (ppm) = 1.46-1.51 (m, 1 H, C5-H), 1.52 (s, 9 H, $C(CH_3)_3$), 1.99-2.05 (m, 1 H, C5-H), 2.21 (dd, ^2J = 16.7 Hz, ^3J = 10.8 Hz, 1 H, C3-H), 2.30-2.43 (m, 1 H, C4-H), 2.63 (ddd, ^2J = 16.7 Hz, ^3J = 5.3 Hz, *J* = 2.3 Hz, 1 H, C3-H), 2.97 (s, 3 H, *N*-CH_3), 3.24 (dd, ^3J = 7.1 Hz, *J* = 2.3 Hz, 2 H, *N*-CH_2), 3.48 (ddd, ^2J = 12.9 Hz, ^3J = 11.5 Hz, ^3J = 4.4 Hz, 1 H, C6-H), 3.85 (ddd, ^2J = 12.9 Hz, ^3J = 5.1 Hz, ^3J = 3.9 Hz, 1 H, C6-H), 6.68 (ddd, ^3J = 6.8 Hz, ^3J = 2.1 Hz, ^4J = 1.0 Hz, 2 H, C_{ortho}-H), 6.71 (tt, ^3J = 7.3 Hz, ^4J = 1.0 Hz, 1 H, C_{para}-H), 7.21-7.25 (m, 2 H, C_{meta}-H).

^{13}C-NMR (90.6 MHz, $CDCl_3$): δ (ppm) = 27.4 (C5), 28.2 (3 × CH_3), 32.1 (C4), 39.4 (C3), 40.0 (*N*-CH_3), 45.6 (C6), 58.1 (*N*-CH_2), 83.2 ($\underline{C}(CH_3)_3$), 112.4 (2 × C_{ortho}), 116.9 (C_{para}), 129.5 (2 × C_{meta}), 149.3 ($C_{ar}N$), 152.7 ($N\underline{C}OO$), 170.6 (C2).

MS (EI): *m/z* (%) = 318 [M^+] (2), 218 (7), 120 (100), 97 (6), 77 (6), 68 (7), 56 (11).

HRMS (EI): calc. for $C_{17}{}^{13}C_1H_{20}N_2O_3$ = 319.1971, found: 319.1965.

2. References

(S1) Alonso, R.; Bach, T. *Angew. Chem. Int. Ed.* **2014**, *53*, in press.; DOI: 10.1002/anie.201310997.

(S2) Curti, C.; Ranieri, B.; Battistini, L.; Rassu, G.; Zambrano, V.; Pelosi, G.; Casiraghi, G.; Zanardi, F. *Adv. Synth.Catal.* **2010**, *352*, 2011–2022.

(S3) Garnier, E. C.; Liebeskind, L.S. *J. Am. Chem. Soc.* **2008**, *130*, 7449–7458.

(S4) Zhang, X.-M.; Mariano, P. S. *J. Org. Chem.* **1991**, *56*, 1655–1660.

(S5) Miyake, Y.; Nakajima, K.; Nishibayashi, Y. *J. Am. Chem. Soc.* **2012**, *134*, 3338–3341.

(S6) Lowry, M. S.; Goldsmith, J. I.; Slinker, J. D.; Rohl, R.; Pascal, R. A.; Malliaras, Jr. G. G.; Bernhard, S. *Chem. Mater.* **2005**, *17*, 5712–5719.

(S7) Marinković, S.; Brulé, C.; Hoffmann, N.; Prost, E.; Nuzillard, J.; Bulach, V. *J. Org. Chem.* **2004**, *69*, 1646–1651.
