# Supplementary material for: Visible-light-induced, Ir-catalyzed reactions of N-methyl-N-((trimethylsilyl)methyl)aniline with cyclic α,β-unsaturated carbonyl compounds
Source: Beilstein J Org Chem. 2014 Apr 17;10:890–6. doi: 10.3762/bjoc.10.86 (PMC3999855; doi:10.3762/bjoc.10.86)
Supplement: File 2 — Tables of all optimization experiments and copies of 1H/13C spectra of PET catalysis products. [file Beilstein_J_Org_Chem-10-890-s002.pdf]

Supporting Information File 2

for

Visible-light-induced, Ir-catalyzed reactions of *N*-methyl-*N*-((trimethylsilyl)methyl)aniline with cyclic α,β-unsaturated carbonyl compounds

Dominik Lenhart and Thorsten Bach*

Address: Department Chemie and Catalysis Research Center (CRC), Technische Universität München, Lichtenbergstr. 4, D-85747 Garching, Germany, Fax: +49-89-28913315

Email: Thorsten Bach* - thorsten.bach@ch.tum.de

* Corresponding author

Tables of all optimization experiments and copies of $^{1}H/^{13}C$ spectra of PET catalysis products

Contents

• 1. Tables of optimization experiments ••S2

1.1 Catalyst screening ••S2

1.2 Solvent screening ••S3

1.3 Catalyst loading screening •••S3

1.4 Screening of equivalents ••S4

1.5 Screening of concentration ••S4

• 2. NMR spectra of PET catalysis products••S5

1. Tables of optimization experiments

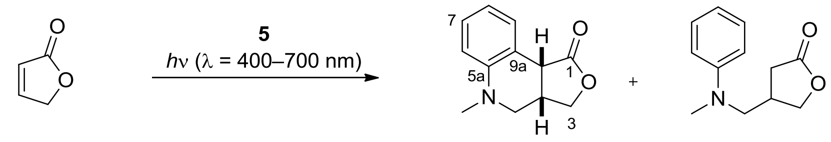

9 10 11

General procedure for PET catalyzed reactions

In a flame dried phototube, all compounds except the photocatalyst were dissolved in the corresponding solvent under an argon atmosphere. The solution was degassed three times via freeze–pump–thaw cycles prior to addition of the catalyst. Irradiation was followed by addition of 10 mL saturated aqueous $NaHCO_3$ solution and 15 mL CH_2Cl_2. The layers were separated and the aqueous layer was extracted with CH_2Cl_2 (2 × 10 mL). The combined organic layers were dried over Na_2SO_4, filtered and the solvent was removed under reduced pressure. The residue was purified by flash chromatography on silica.

1.1 Catalyst screening

conc: 0.1 M, *T* = rt, solvent = CH_2Cl_2, catalystloading = 5 mol %, **9**/**5** = 1:1.5

catalyst	*t* [h]	Y(**10**) [%]
$[Ru(bpy)_3Cl_2]*6H_2O$	22	30[a]
$[Ir(ppy)_2(bpy)]BF_4$	22	42
$[Ir(ppy)_2(dtbbpy)]BF_4$	24	46
$[Ir((dF)(CF_3)ppy)_2(bpy)]PF_6$	24	37
$[Ir((dF)(CF_3)ppy)_2(dtbbpy)]PF_6$	24	26[b]
Eosin Y * TBA_2	24	8[c]

[a]: in CH_2Cl_2/MeOH 9:1, 9% of **11**, [b]: 80% conversion, [c]: 85% conversion.

1.2 Solvent screening

conc. = 0.1 M, T = rt, catalyst: $[Ir(ppy)_2(dtbbpy)]BF_4$, catalystloading = 5 mol %, **9**/**5** = 1:1.5

solvent	*t* [h]	Y [%]	**10**:**11**
MeOH	5	40	>99:1
DMF	5	31	2.4:1
DMSO	5	40	4.7:1
DMA	5	25	4:1
MeCN/H_2O 3:1	5	41	20:1
MeCN	24	41	9.3:1
acetone	24	41	>99:1
toluene	24	12[a]	>99:1
THF	24	21	9.5:1
1,4 dioxane	24	21[b]	>99:1
CH_2Cl_2/MeOH 9:1	24	35[c]	>99:1

[a]: c = 10 mM, [b]: c = 10 mM, 92% conversion, [c]: 91% conversion

1.3 Catalyst loading screening

conc. = 0.1 M, T = rt, catalyst: $[Ir(ppy)_2(dtbbpy)]BF_4$, solvent = MeOH, **9**/**5** = 1:1.5

catload [mol %]	*t* [h]	Y(**10**) [%]
1	5	47
2.5	5	49
5	5	40
10	5	14

1.4 Screening of equivalents

conc. = 0.1 M, *T* = rt, catalyst: $[Ir(ppy)_2(dtbbpy)]BF_4$, catalystloading = 2.5 mol %, solvent = MeOH

**9**:**5**	*t* [h]	Y(**10**) [%]
1:1	5	37
1.5:1	6	47
5:1	6	47
10:1	5	14

1.5 Screening of concentration

T = rt, catalyst: $[Ir(ppy)_2(dtbbpy)]BF_4$, catalystloading = 2.5 mol %, **9**/**5** = 1:1.5, solvent = MeOH

concentration [M]	*t* [h]	Y(**10**) [%]
0.1	5	49
0.05	5	36
0.025	5	37

2. NMR spectra of PET catalysis products

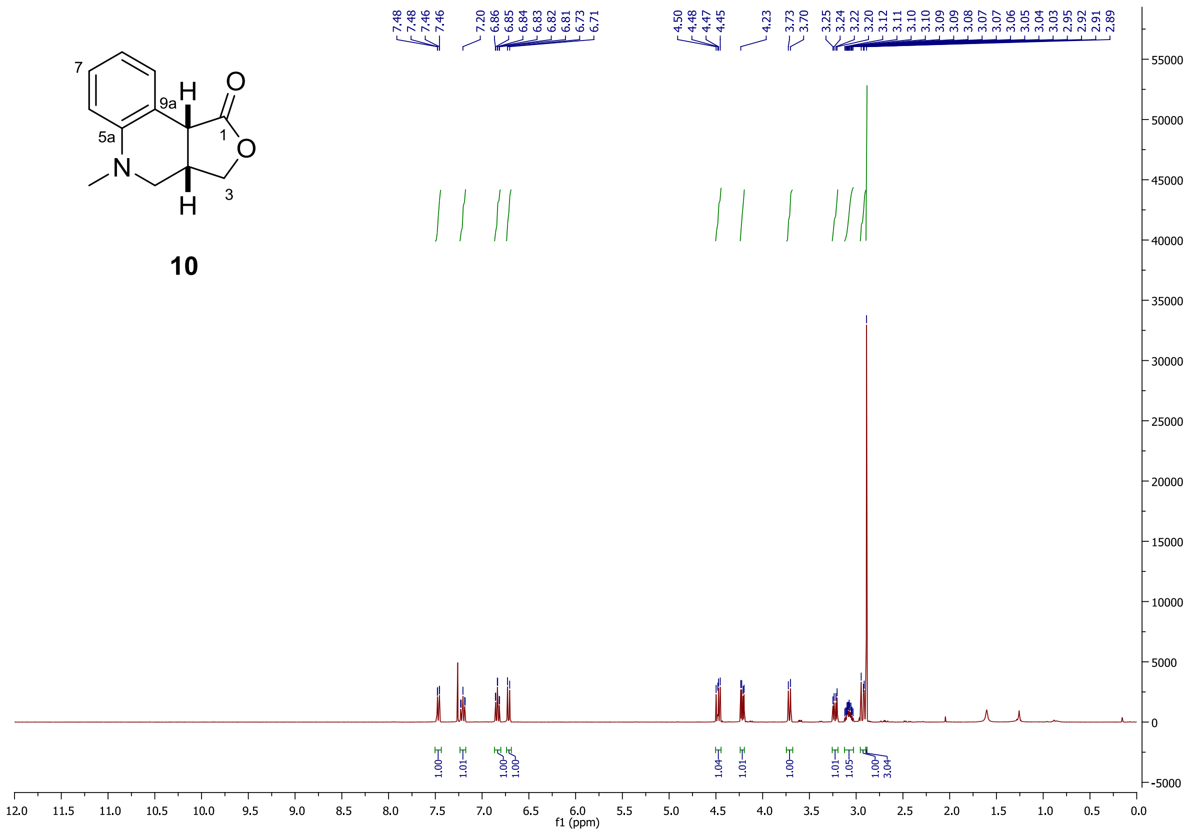

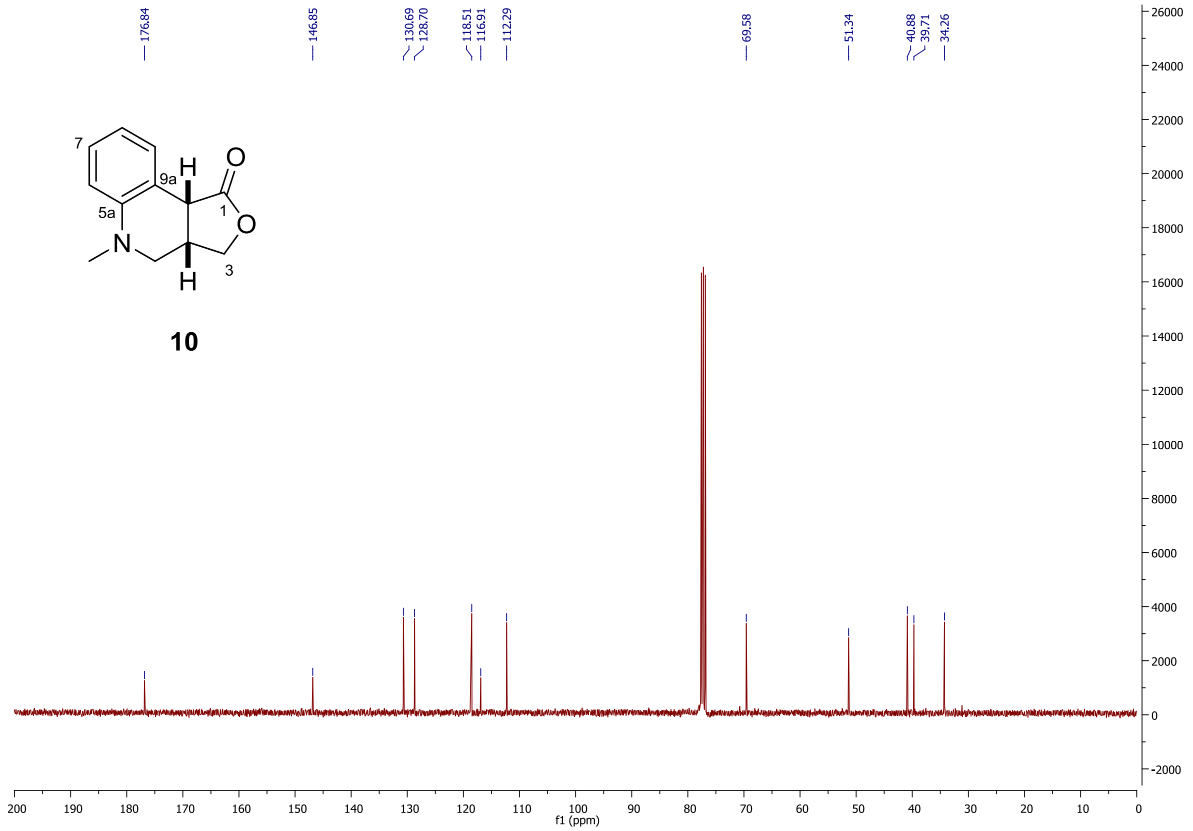

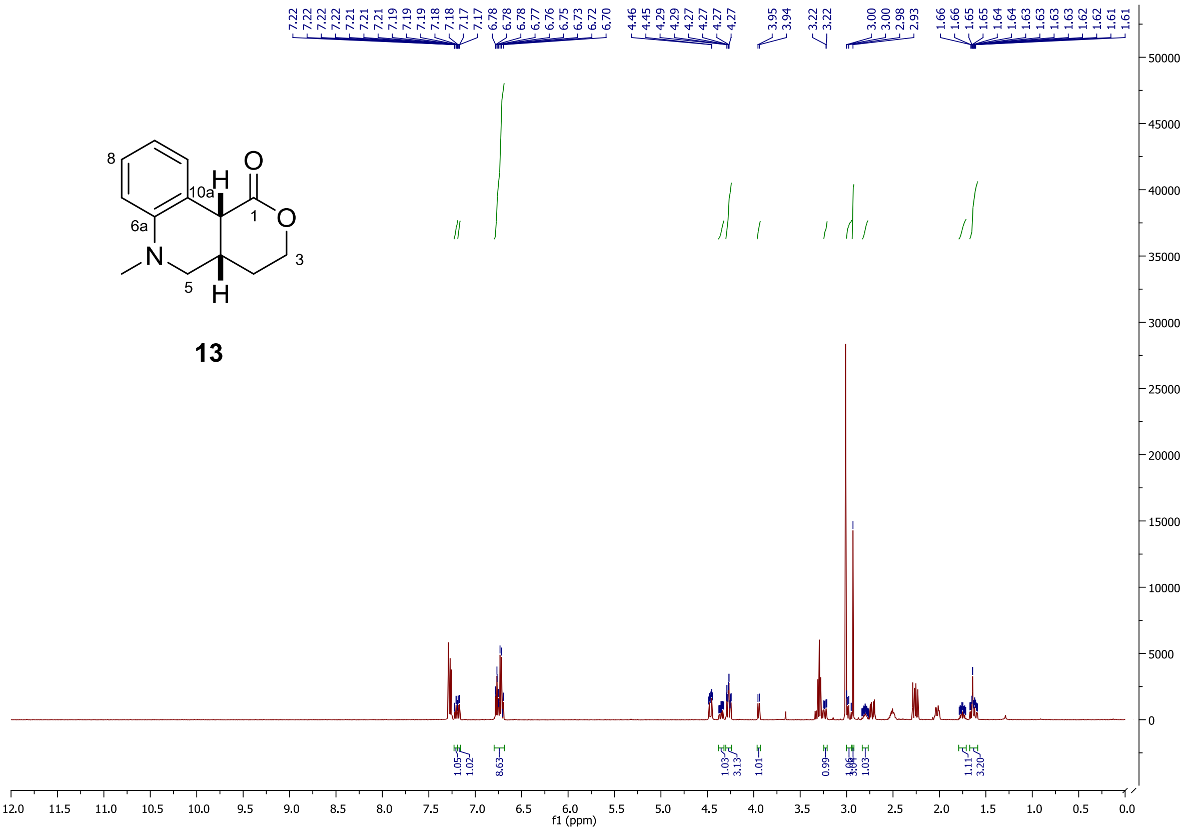
7.22 7.22 7.22 7.22 7.21 7.21 7.19 7.19 7.19 7.18 7.17 7.17 6.78 6.78 6.77 6.76 6.75 6.73 6.72 6.70 4.46 4.45 4.29 4.29 4.27 4.27 4.27 4.27 3.95 3.94 3.22 3.22 3.00 3.00 2.98 2.93 1.66 1.66 1.65 1.65 1.64 1.64 1.63 1.63 1.63 1.62 1.61 1.61
8
10a
H
O
1
O
6a
N
3
5
H
13
1.05
1.02
8.63
1.03
3.13
1.01
0.99
1.03
1.11
3.20
f1 (ppm)

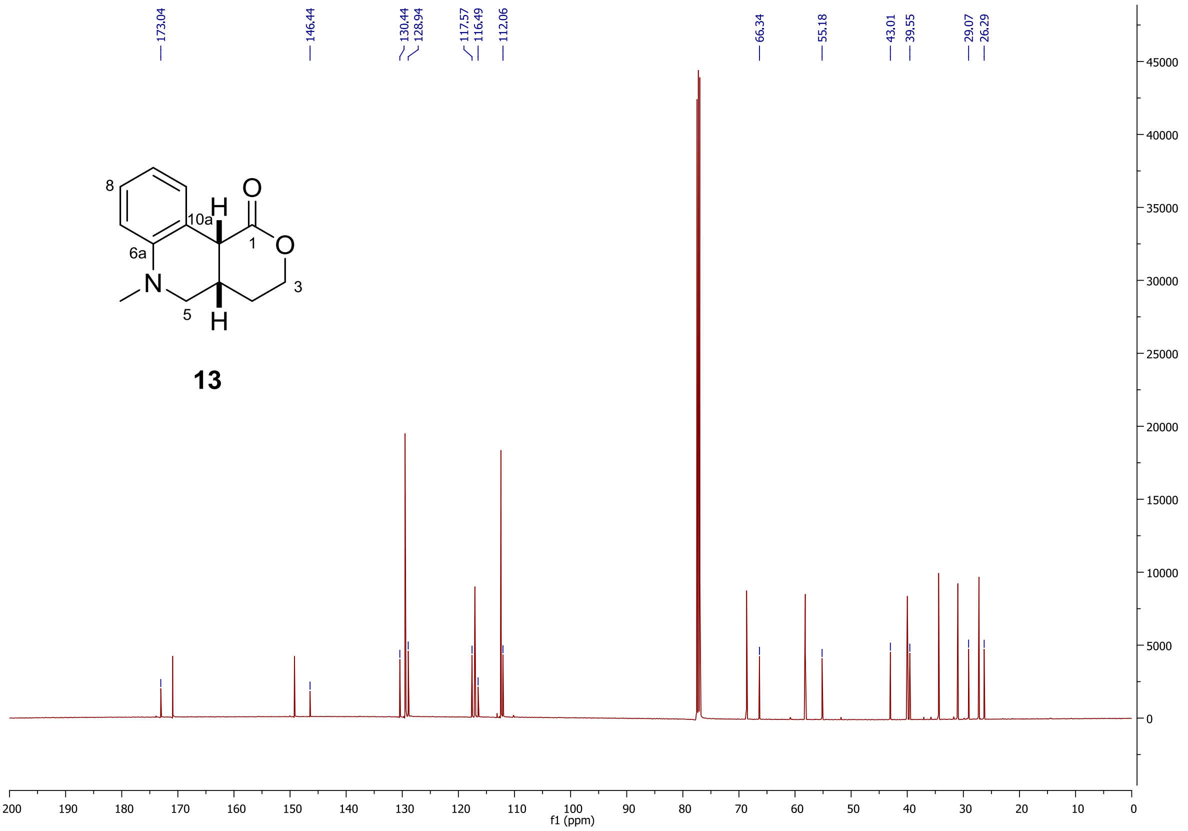
173.04
146.44
130.44
128.94
117.57
116.49
112.06
66.34
55.18
43.01
39.55
29.07
26.29
8
10a
H
O
1
O
6a
N
3
5
H
13
f1 (ppm)

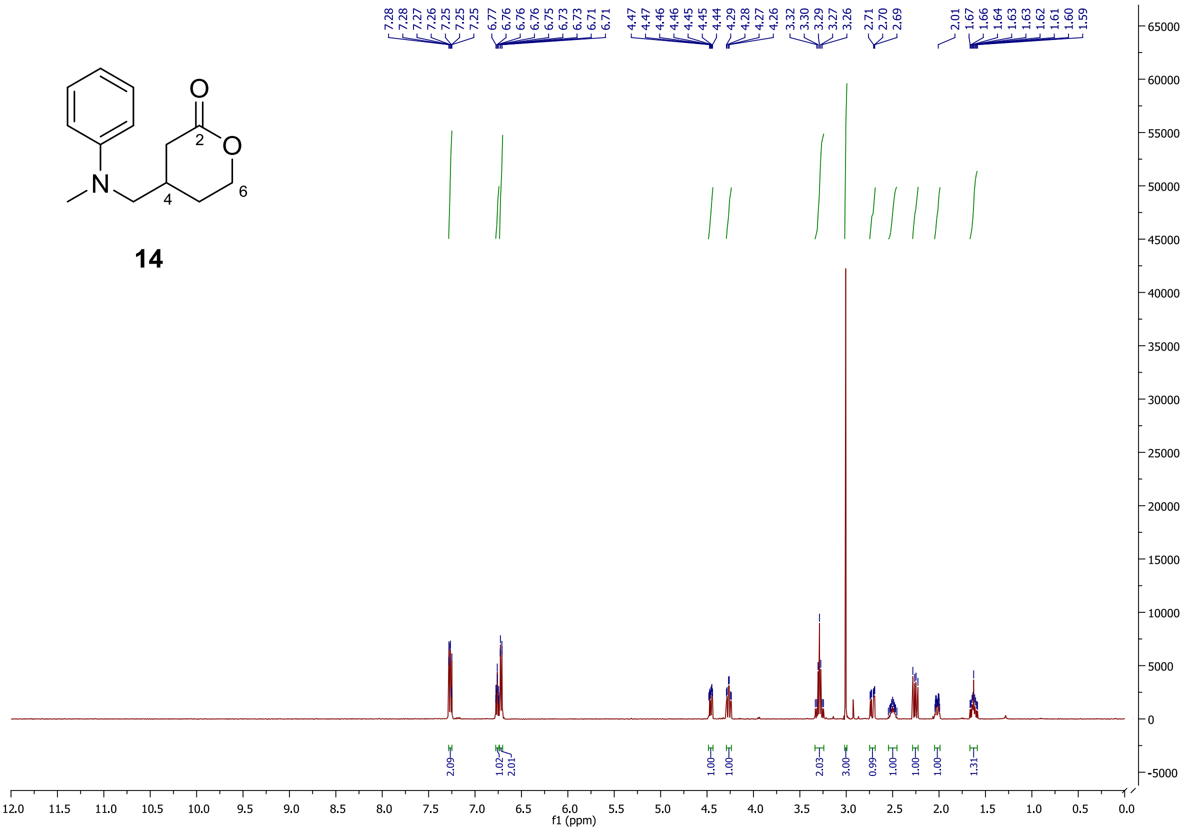
7.28 7.28 7.27 7.26 7.25 7.25 7.25 6.77 6.76 6.76 6.76 6.75 6.73 6.73 6.71 6.71 4.47 4.47 4.46 4.46 4.45 4.45 4.44 4.29 4.28 4.27 4.26 3.32 3.30 3.29 3.27 3.26 2.71 2.70 2.69 2.01 1.67 1.66 1.64 1.63 1.63 1.62 1.61 1.60 1.59
O
2
O
N
4
6
14
2.09 1.02 2.01 1.00 1.00 2.03 3.00 0.99 1.00 1.00 1.00 1.31
f1 (ppm)

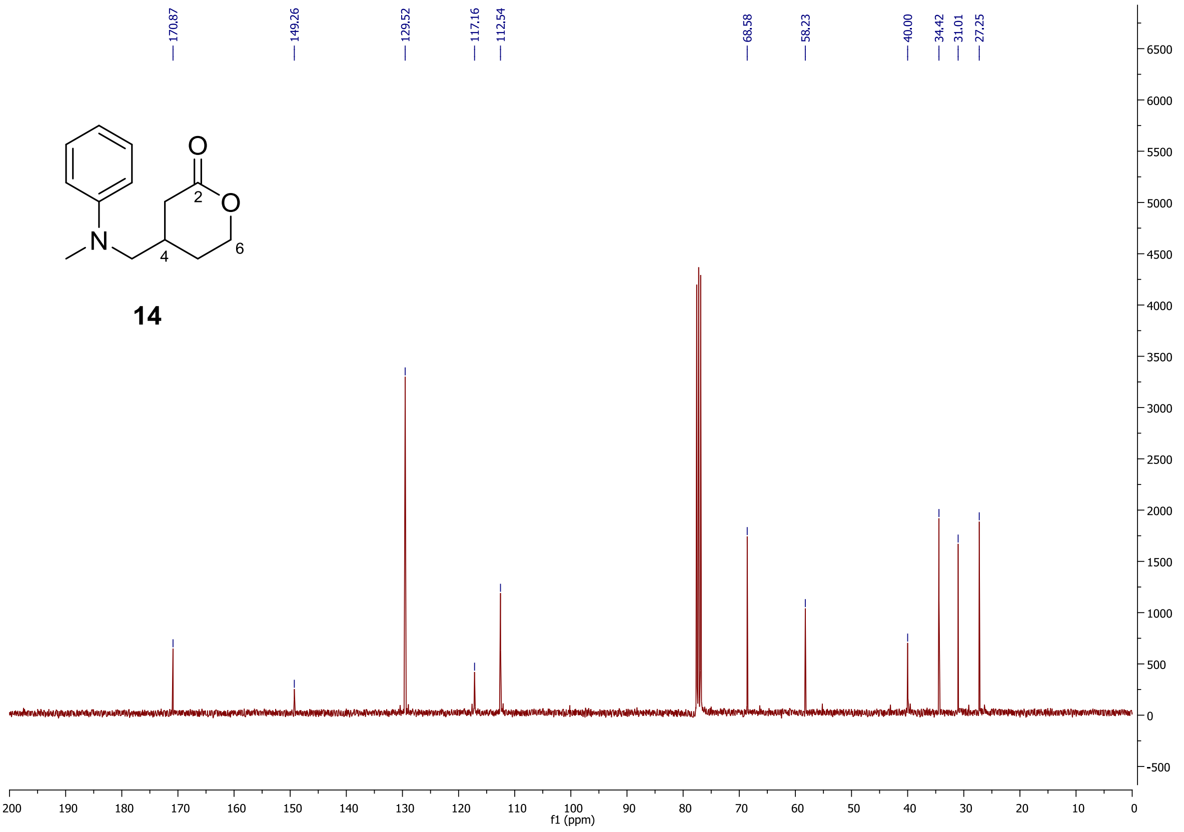
170.87
149.26
129.52
117.16
112.54
68.58
58.23
40.00
34.42
31.01
27.25
O
2
O
N
4
6
14
f1 (ppm)

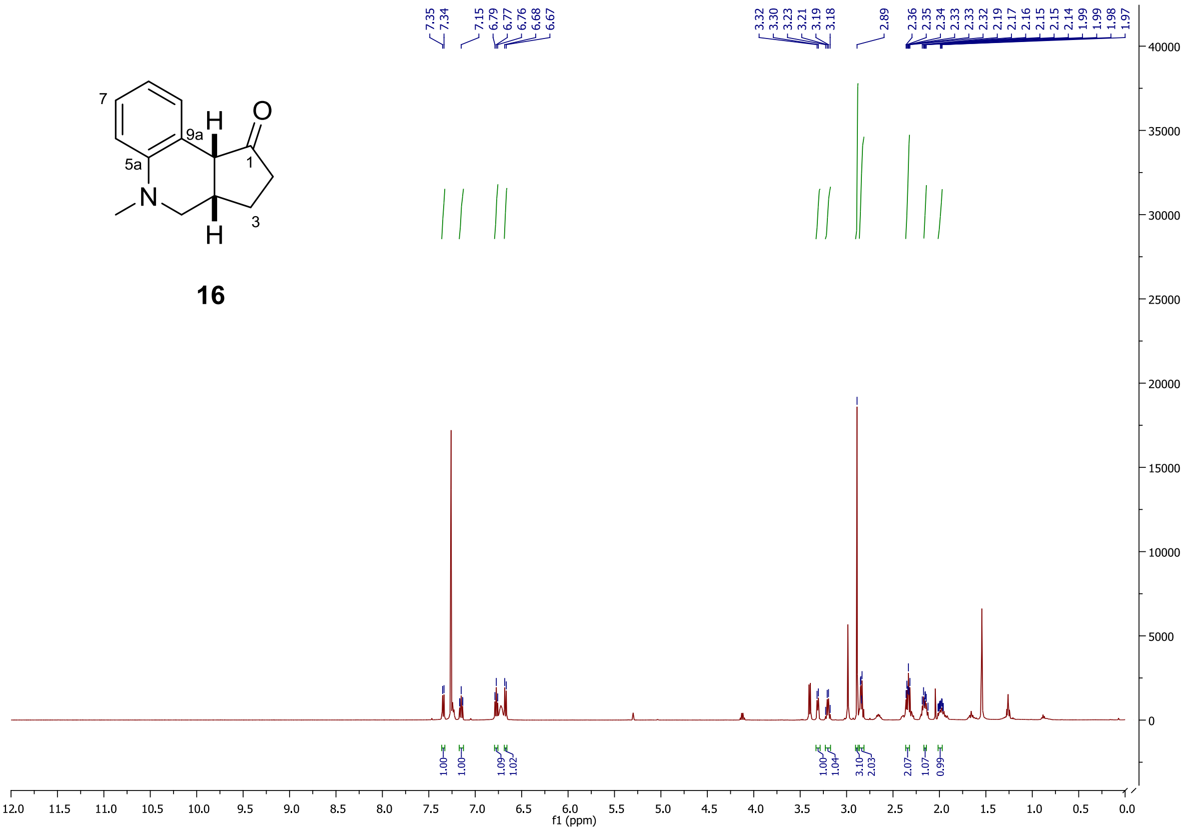
7
9a
5a
1
N
3
H
O
7.35
7.34
7.15
6.79
6.77
6.76
6.68
6.67
3.32
3.30
3.23
3.21
3.19
3.18
2.89
2.36
2.35
2.34
2.33
2.33
2.32
2.19
2.17
2.16
2.15
2.14
1.99
1.99
1.98
1.97
1.00
1.00
1.09
1.02
1.00
1.04
3.10
2.03
2.07
1.07
0.99
f1 (ppm)

16

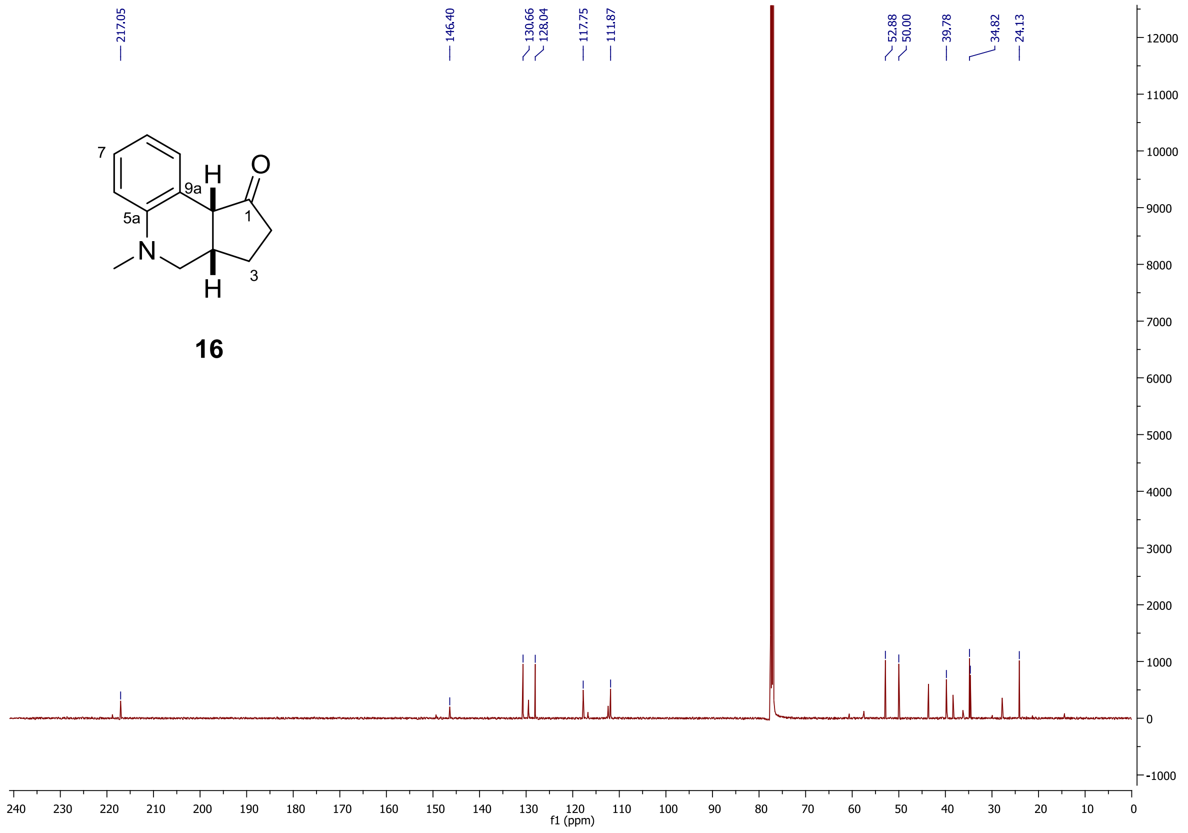
217.05
146.40
130.66
128.04
117.75
111.87
52.88
50.00
39.78
34.82
24.13
f1 (ppm)

16

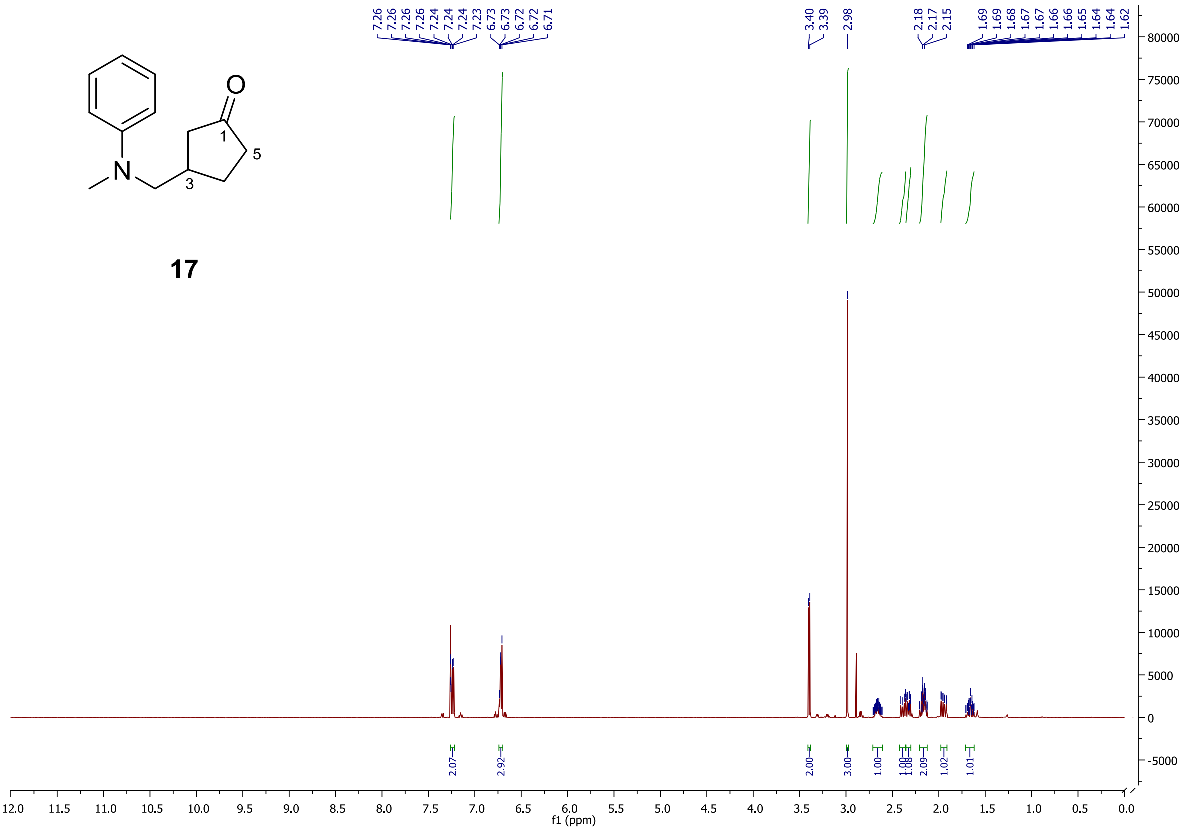

7.26 7.26 7.26 7.26 7.24 7.24 7.24 7.23 6.73 6.73 6.72 6.72 6.71
3.40 3.39 2.98
2.18 2.17 2.15
1.69 1.69 1.68 1.67 1.67 1.66 1.66 1.65 1.64 1.64 1.62
O
1
5
N
3
2.07 2.92 2.00 3.00 1.00 1.00 1.08 2.09 1.02 1.01
f1 (ppm)

17

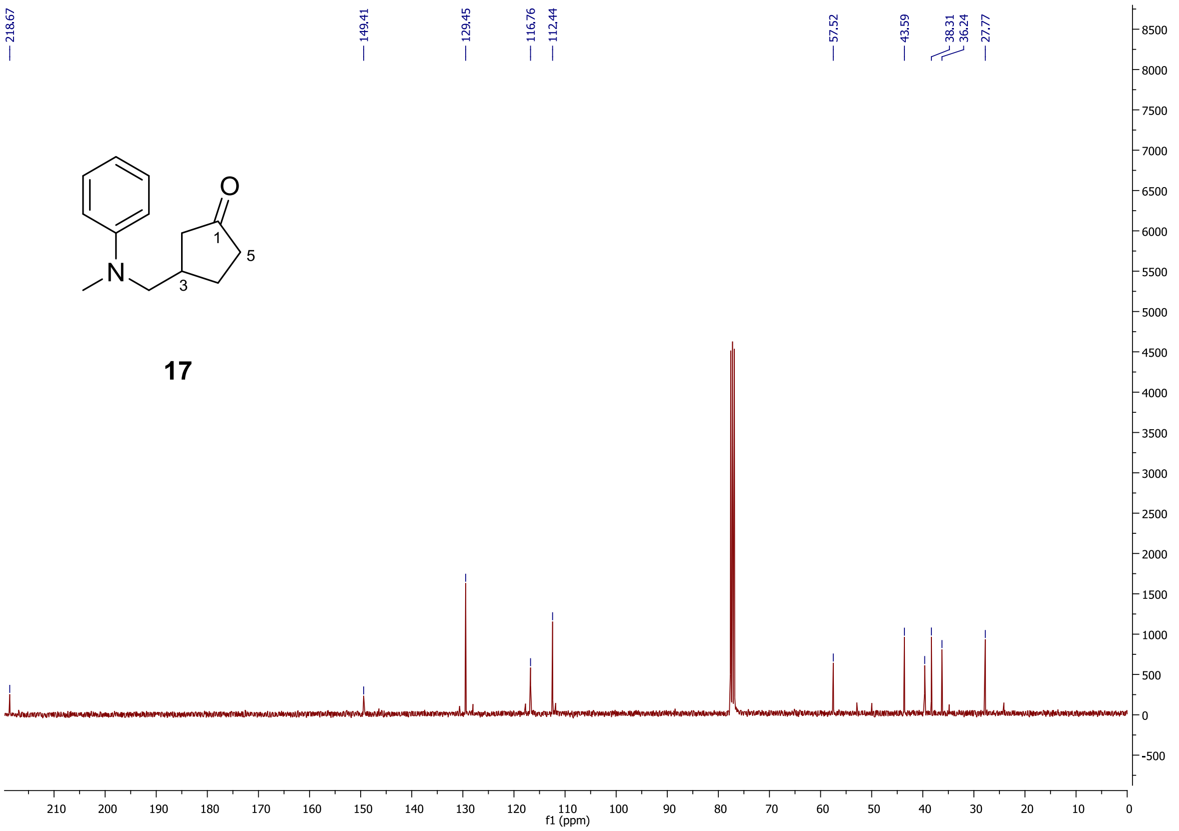

218.67
149.41
129.45
116.76
112.44
57.52
43.59
38.31
36.24
27.77
O
1
5
N
3
f1 (ppm)

17

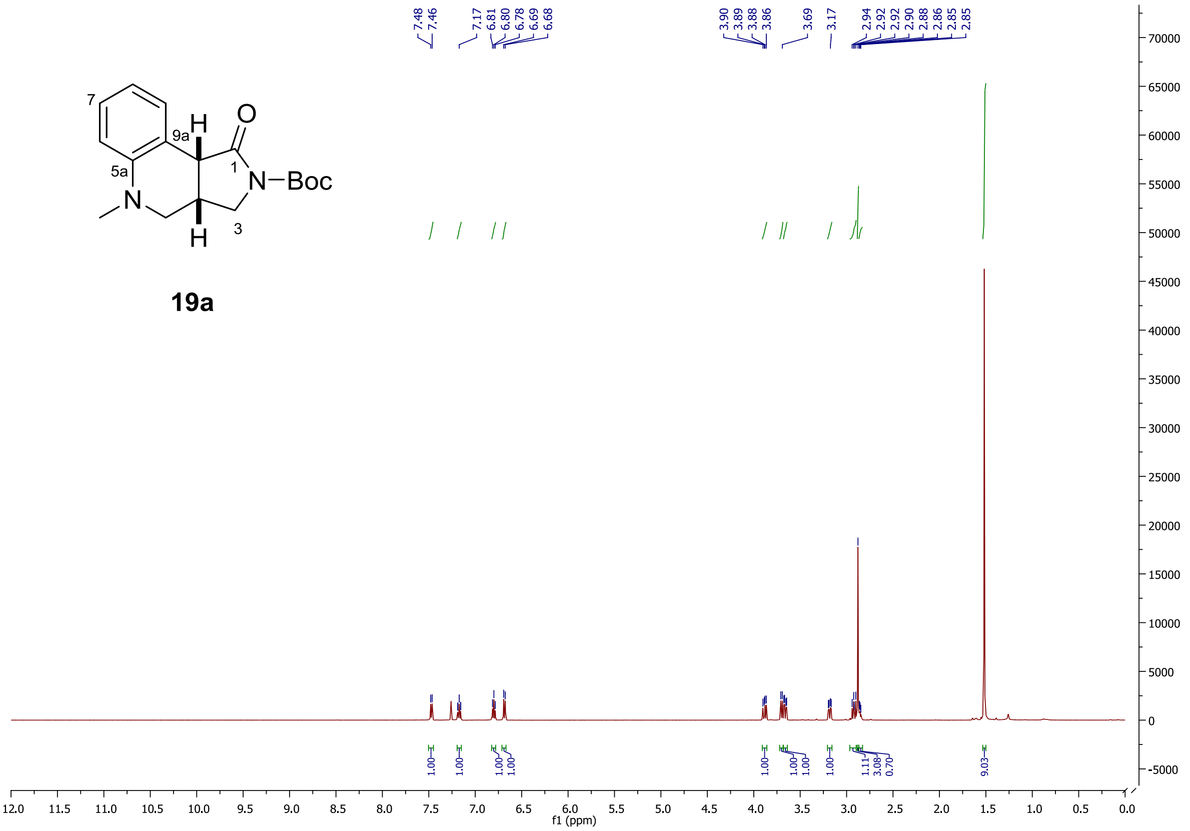
7.48
7.46
7.17
6.81
6.80
6.78
6.69
6.68
3.90
3.89
3.88
3.86
3.69
3.17
2.94
2.92
2.92
2.90
2.88
2.86
2.85
7
9a
H
O
5a
1
N
N−Boc
H
3
19a
1.00
1.00
1.00
1.00
1.00
1.00
1.00
1.00
1.11
3.08
0.70
9.03
f1 (ppm)

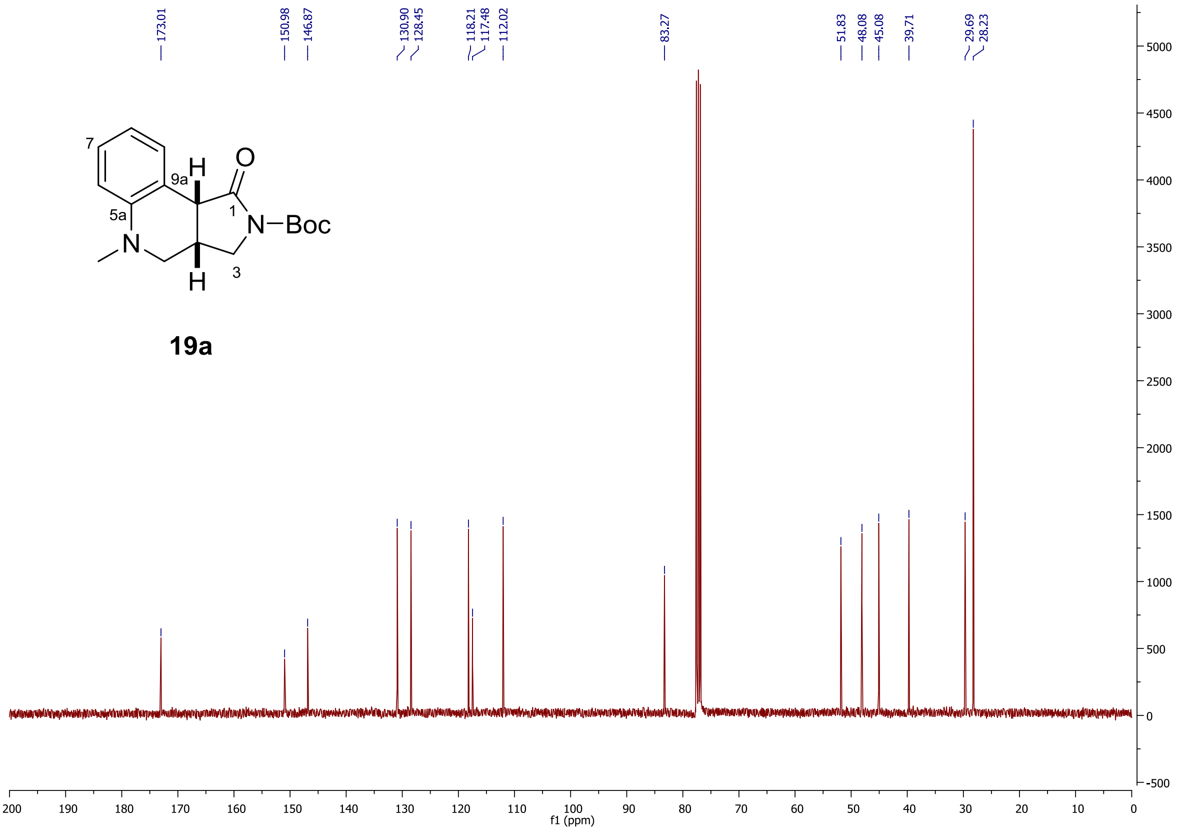
173.01
150.98
146.87
130.90
128.45
118.21
117.48
112.02
83.27
51.83
48.08
45.08
39.71
29.69
28.23
7
9a
H
O
5a
1
N
N−Boc
H
3
19a
f1 (ppm)

19b

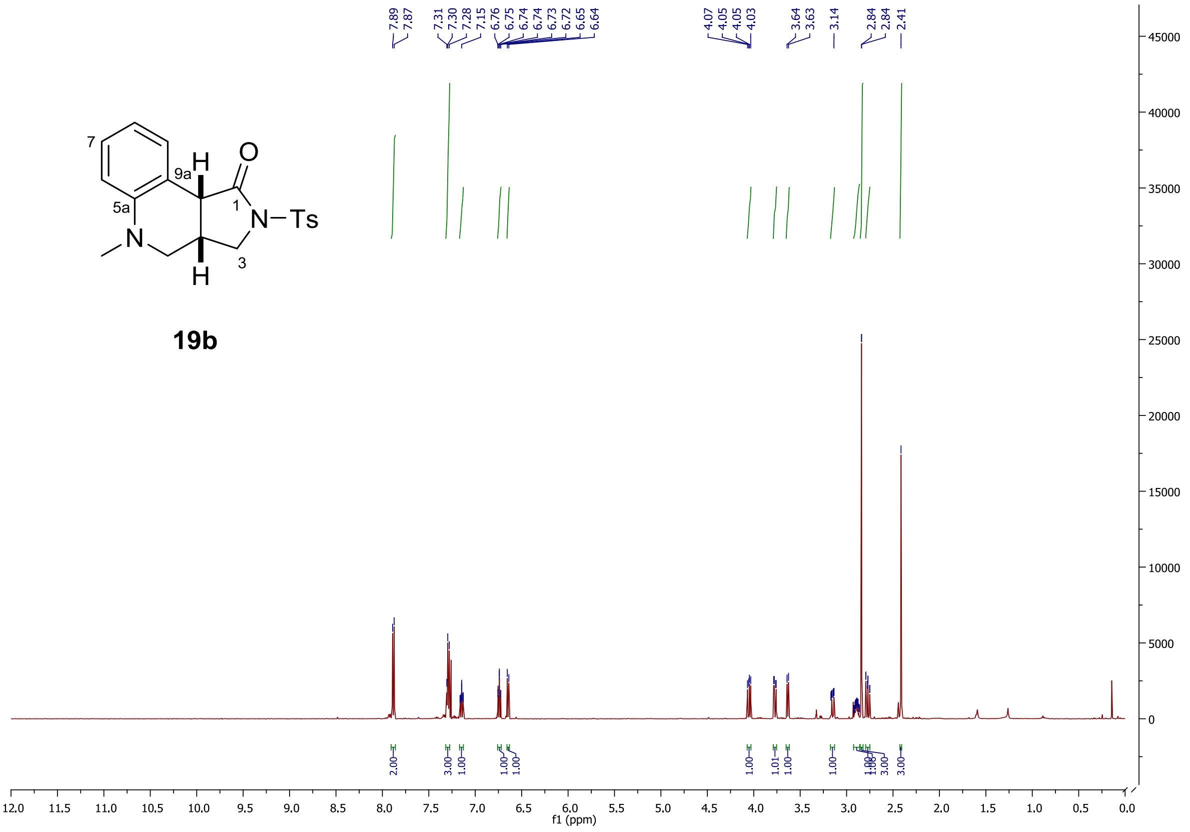

7.89
7.87
7.31
7.30
7.28
7.15
6.76
6.75
6.74
6.74
6.73
6.72
6.65
6.64
4.07
4.05
4.05
4.03
3.64
3.63
3.14
2.84
2.84
2.41
f1 (ppm)

19b

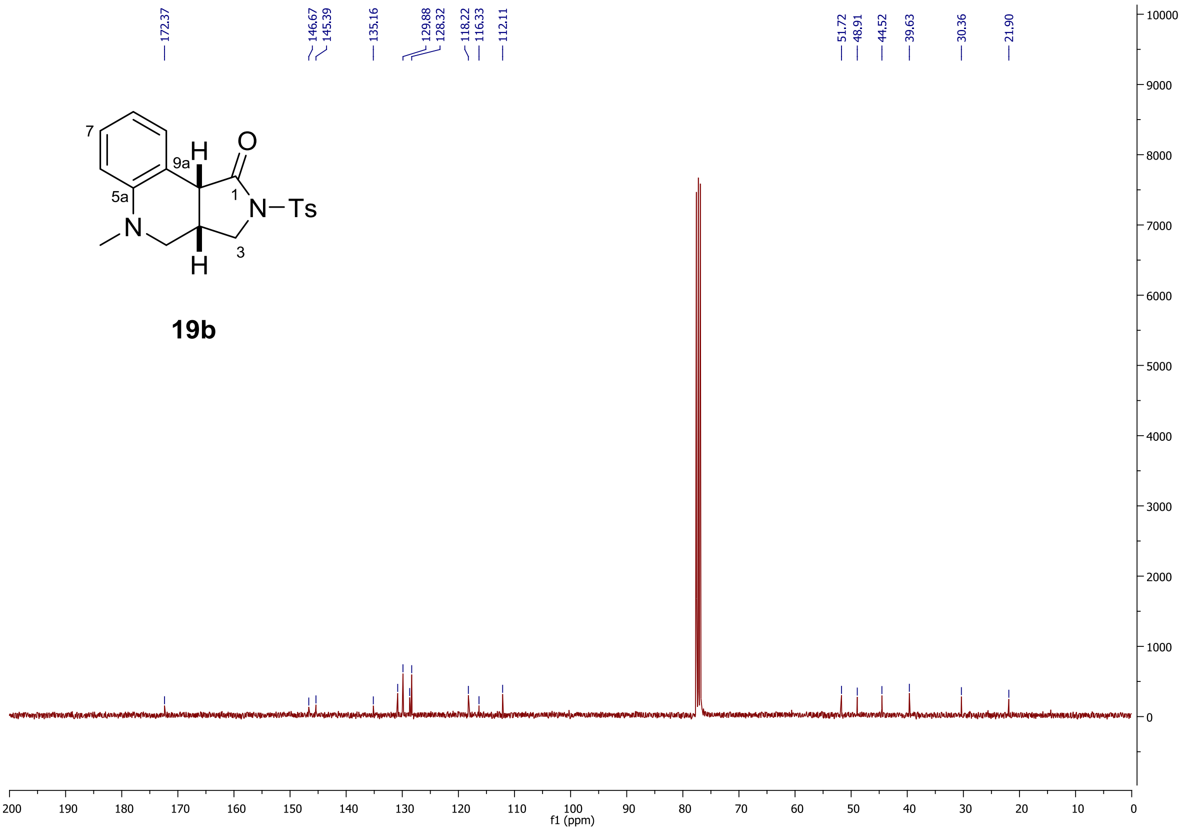

172.37
146.67
145.39
135.16
129.88
128.32
118.22
116.33
112.11
51.72
48.91
44.52
39.63
30.36
21.90
f1 (ppm)

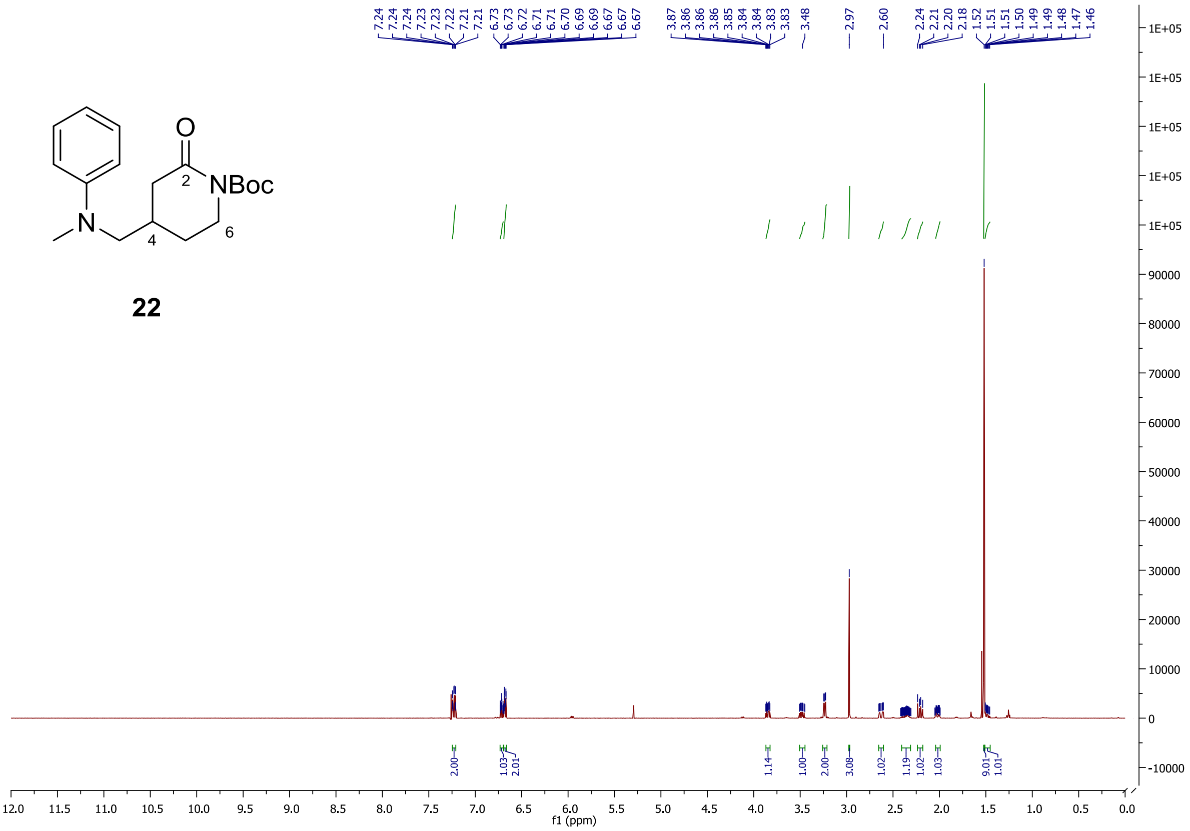
7.24
7.24
7.24
7.23
7.23
7.22
7.21
7.21
6.73
6.73
6.72
6.71
6.71
6.70
6.69
6.69
6.67
6.67
6.67
3.87
3.86
3.86
3.86
3.85
3.84
3.84
3.83
3.83
3.48
2.97
2.60
2.24
2.21
2.20
2.18
1.52
1.51
1.51
1.50
1.49
1.49
1.48
1.47
1.46
O
2
NBoc
N
4
6
22
2.00
1.03
2.01
1.14
1.00
2.00
3.08
1.02
1.19
1.02
1.03
9.01
1.01
f1 (ppm)

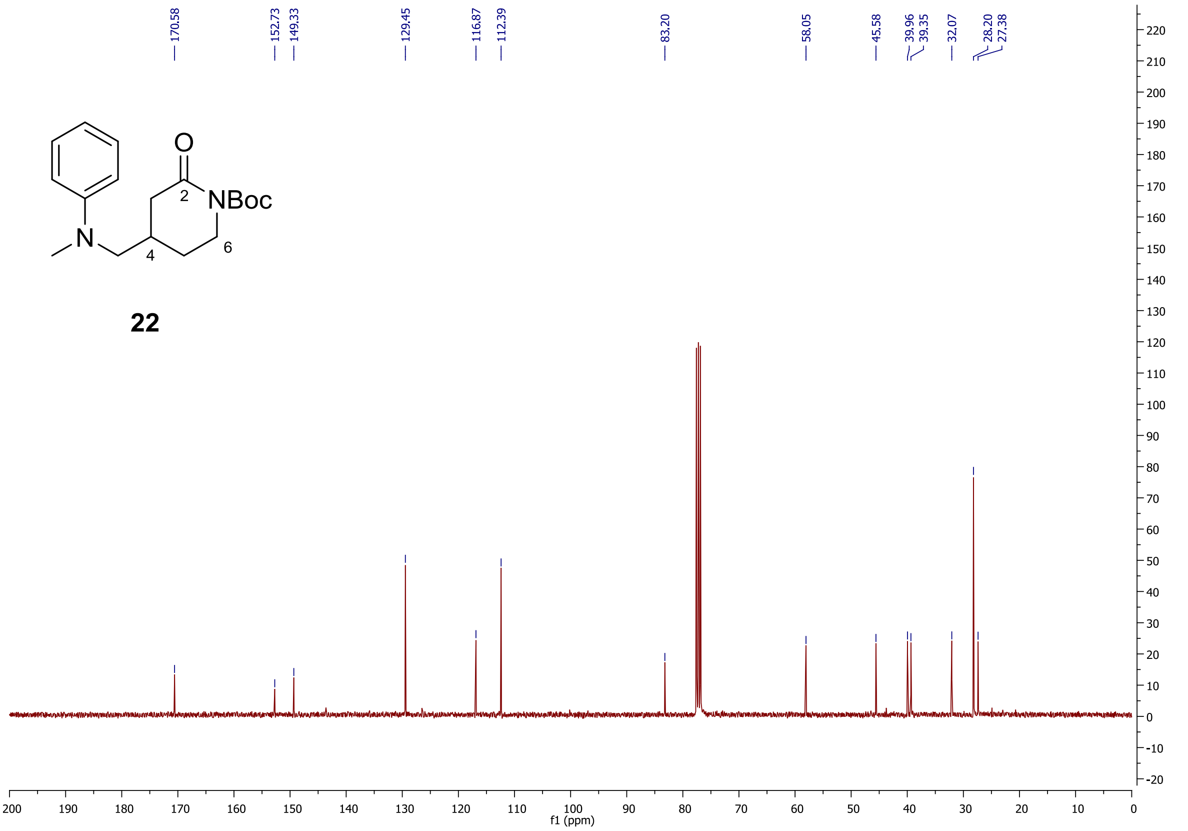
170.58
152.73
149.33
129.45
116.87
112.39
83.20
58.05
45.58
39.96
39.35
32.07
28.20
27.38
O
2
NBoc
N
4
6
22
f1 (ppm)
